# 5-Aminoisophthalate-based kojic acid-appended bis-1,2,3-triazole: a fluorescent chemosensor for Cu^2+^ sensing and *in silico* study†

**DOI:** 10.1039/d4ra02372b

**Published:** 2024-07-03

**Authors:** Sachin Kumar, Bajrang Lal, Gurleen Singh, Muskan, Ram Kumar Tittal, Jandeep Singh, Ghule Vikas D., Renu Sharma

**Affiliations:** a Department of Chemistry, National Institute of Technology Kurukshetra Haryana 136119 India rktittaliitd@nitkkr.ac.in +91-1744-233-542; b School of Applied Sciences, Om Sterling Global University Hisar Haryana 125001 India; c School of Chemical Engineering and Physical Sciences, Lovely Professional University Phagwara Punjab 144411 India singhjandeep@gmail.com; d Department of Chemistry, University of Delhi Delhi 110007 India

## Abstract

A new, easy-to-prepare, and highly selective fluorescent chemosensor, *i.e.*, 5-aminoisophthalate-based kojic acid-appended bis-1,2,3-triazole, was synthesized from an alkyne of 5-aminoisophthalic acid and azido-kojic acid using Cu(i)-catalyzed click chemistry and then successfully characterized. The alkyne structure of 5-aminoisophthalic acid, 1, was supported by the single-crystal X-ray crystallographic data. The fluorescent probe 3 was found to be highly selective for Cu^2+^ ions supported by the Job's plot with a stoichiometric ligand : metal ratio of 2 : 1, exhibiting almost a two-fold enhancement in the emission intensity upon the addition of Cu^2+^ ions (0–25 μM) with a detection limit of 8.82 μM. A comparison with LODs from previously developed chemosensors for Cu^2+^ ions was also conducted. Reversibility analysis indicated that probe 3 could be used as both a reusable sensor and as a scavenger of copper ions. DFT calculations with the basis sets B3LYP/6-311G(d,p) and LanL2DZ were employed for geometrical optimizations of structures of the alkyne 1, azide 2, probe 3, and complex 3.Cu^2+^. Hirshfeld surface analysis revealed significant intermolecular interactions in compound 1. Additionally, molecular docking for the antimicrobial activity showed the better antibacterial efficacy of probe 3.

## Abstract 2

A new, easy-to-prepare, and highly selective 5-aminoisophthalate and kojic acid linked bis-1,2,3-triazole chemosensor was synthesized from an alkyne of 5-aminoisophthalic acid and azido-kojic acid by CuAAC reaction and fully characterized.

## Graphical abstract

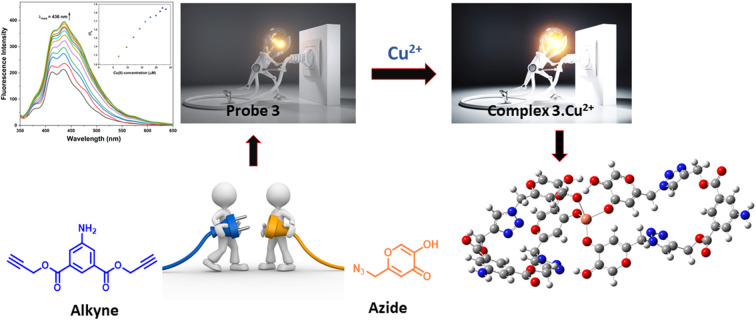

## 1. Introduction

In the realm of chemical and biological research, the development of innovative fluorescent probes for the selective detection of metal ions and exploration of potential antimicrobial agents with desired characteristics have become paramount. Among these, exploring diverse heterocyclic scaffolds has attracted significant attention owing to their versatile applications in various fields including medicinal chemistry and materials science.^1,2^ Due to their unique electronic and structural properties, triazoles, a class of five-membered heterocyclic compounds, have emerged as intriguing candidates. Amongst the various fluorescent chemosensors available, triazoles, obtained *via* CuAAC through a ‘click’ methodology,^3–6^ which is considered the ideal synthetic route to form 5-aminoisophthalate-based kojic acid-appended 1,4-disubstituted bis-1,2,3-triazole derived from natural compounds, offer unique advantages due to their biocompatibility and eco-friendly nature. Kojic acid, a natural product obtained from fungi, has attracted significant attention for its diverse applications in medicine and materials science.^7–10^ The application of the synthesized 5-aminoisophthalate-based kojic acid-appended 1,4-disubstituted bis-1,2,3-triazole as a sensing platform for heavy and transition metal ions is of specific interest after prior research in related fluorescent and biologically significant hybrid compounds.^11,12^

The significant increase in the accumulation of metal ions beyond acceptable levels has underscored the urgent need to investigate effective and robust research substitutes for their immediate recognition, even at low concentrations.^13,14^ The focus on metal cation detection arises from their extensively documented harmful effects on terrestrial and aquatic ecosystems,^15,16^ making their qualitative and quantitative identification imperative.^17^ As the third-most-common heavy metal ion in the human body, bivalent copper (Cu^2+^) is an essential ion that plays several roles in biological processes. Functioning as a catalytic cofactor for metalloenzymes,^18^ it plays a pivotal role in many critical physiological functions. However, an intricate balance of metal ions is crucial, as the dysregulation of Cu^2+^ ions can lead to oxidative stress and associated health complications. Copper deficiency in the human body is implicated in several serious conditions, such as Alzheimer's disease,^19,20^ and Wilson's disease.^21^

On the other hand, elevated concentrations of copper pose risks to vital organs, particularly the liver and kidneys.^22^ Therefore, it is crucial to maintain copper homeostasis, which calls for sophisticated regulatory mechanisms. Environmental pollution resulting from the accumulation of Cu^2+^ ions through industrial discharge is also a matter of concern.^23^

The research community is compelled to explore cutting-edge approaches for the efficient and selective detection of this metal, driven by various factors. Moreover, the significance of optical technologies has grown substantially owing to their sensitive responses, practical feasibility, economic viability, and abundant biocompatibility. These attributes collectively enable their extension to real-time applications, facilitating the monitoring of copper levels in biological and environmental contexts.

This research study explored the synthesis of 5-aminoisophthalate-based kojic acid-appended 1,4-disubstituted bis-1,2,3-triazole, and investigated its promising potential, particularly in metal ion sensing and *in silico* exploration of the synthesized probe's potential antimicrobial properties. The focus of this study revolved around the strategic incorporation of 5-amino isophthalic acid and kojic acid as crucial precursors in the synthesis process. This choice is not arbitrary but stems from their distinctive reactivity, *e.g.*, using 5-amino isophthalic acid, with its amino functionality and aromatic character, is anticipated to introduce desirable electronic properties to the triazole backbone. Concurrently, the inclusion of kojic acid, renowned for its chelating capabilities and excellent coordination properties,^24,25^ can add an element of selectivity and sensitivity to the synthesized chemosensor, particularly in the context of metal ion recognition. Molecular docking, a powerful computational technique, was used to investigate the interaction between the probe and selected microbial targets. This dual-purpose investigation sheds light on the probe's ability to selectively detect Cu^2+^ ions and its potential as an antimicrobial agent. The outcomes of this research hold promise for contributing to the advancement of fluorescence-based sensing technologies and the discovery of novel antimicrobial agents with potential therapeutic applications.

Considering our experience in the synthesis of hybrid compounds linked with 1,2,3-triazole/fluorophoric groups for designing pharmacologically important compounds or chemosensors for selectively detecting metal ions/biothiols,^26,27^ we herewith present for the first time a 5-aminoisophthalate-based kojic acid-appended 1,4-disubstituted bis-1,2,3-triazole as a chemosensor for the selective detection of Cu^2+^ ions in the presence of other metal ions, such as Pb^2+^, Cr^3+^, Mn^2+^, Fe^2+^, Co^2+^, Ni^2+^, Cu^2+^, Zn^2+^, Cd^2+^, Hg^2+^, Na^+^, K^+^, Mg^2+^, Ca^2+^, and Ba^2+^.

## 2. Experimental section

### 2.1 Materials and methods

All chemicals utilized in this study were procured from reputable suppliers. Specifically, 5-aminoisophthalic acid, K_2_CO_3_, NaN_3_, and various metal chlorides (Cr^3+^, Mn^2+^, Fe^2+^, Co^2+^, Ni^2+^, Cu^2+^, Zn^2+^, Cd^2+^, Hg^2+^, Na^+^, K^+^, Mg^2+^, Ca^2+^, and Ba^2+^) and lead acetate: Pb^2+^ were sourced from Sigma Aldrich. Kojic acid was acquired from TCI Chemicals, while propargyl bromide was obtained from Avra. Quinine sulfate served as the reference standard for determining the fluorescence quantum yields. All solvents were purchased from Loba Chemie. NMR spectra (^1^H and ^13^C) were recorded using a Bruker Avance (II) 500 MHz instrument in CDCl_3_ and DMSO-*d*_6_. High-resolution mass spectrometry (HRMS) analyses were performed using an electrospray mass spectrometer. The Fourier transform infrared (FTIR) spectra were recorded using a Shimadzu IR Affinity-1S spectrophotometer. Sensing studies were conducted using SHIMADZU UV-1900 and PerkinElmer FL 6500 spectrophotometers.

### 2.2 Synthesis of di(prop-2-yn-1-yl) 5-aminoisophthalate, 1

The alkyne synthesis was commenced by treating 5 mmol of 5-aminoisophthalic acid with 11 mmol propargyl bromide in 10 mL of DMF, utilizing 25 mmol anhydrous potassium carbonate as a base. The reaction, monitored by TLC, proceeded over 10 h. After the reaction was finished, ice-cold water was used to quench the mixture, yielding a white solid. The precipitate was thoroughly washed with chilled water and subsequently dried in a vacuum. Purification of the alkyne involved passing it through a short silica gel column using ethyl acetate : hexane (2 : 8, v/v) as the eluent. This purification process isolated alkyne 1 as a white solid with an impressive 88% yield.

Appearance: white solid; yield: 88%; mpt: 90–92 °C; FTIR (*ν*_max_ cm^−1^): 3421, 3350, 3305, 3269, 2129, 1716, 1697, 1600, 1444, 1330, 1234, 1132, 1016, 906, 758, 553; ^1^H NMR (500 MHz, DMSO-*d*_6_) *δ* = 7.64(t, *J* = 1.55, 1H), 7.44 (d, *J* = 1.50, 2H), 5.83 (s, 2H), 4.95 (d, *J* = 2.35, 4H), 3.62 (t, *J* = 2.45, 2H); ^13^C NMR (126 MHz, CDCl_3_) *δ* = 165.27 (C), 147.02 (C), 130.99 (C), 121.11 (CH), 120.31 (CH), 77.67 (C), 75.35 (CH), 52.82 (CH_2_); HRMS (ESI^+^): [M + H]^+^ calculated for C_14_H_11_NO_4_^+^ is 258.0761, found 258.0767.

**Fig. 1:**
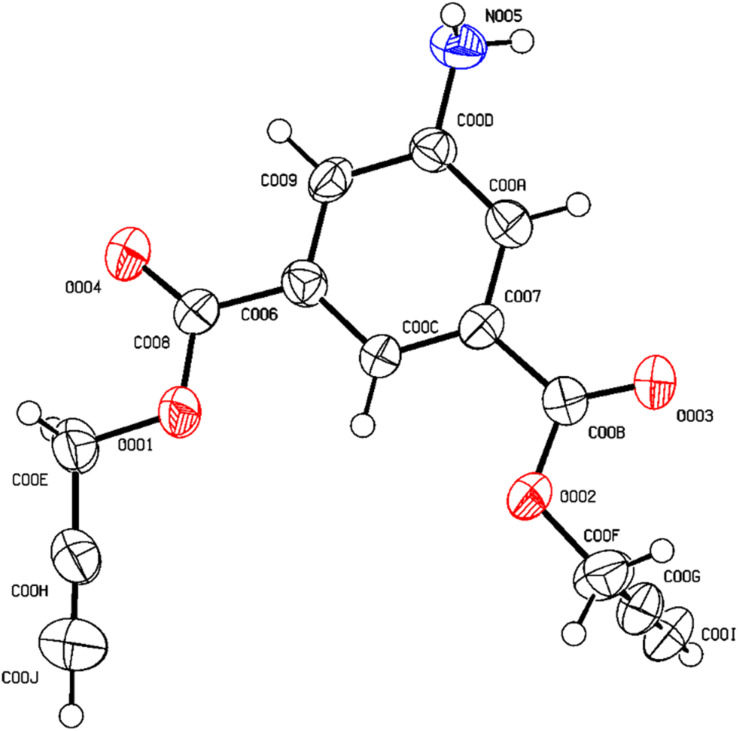
ORTEP diagram of compound 1.

**Fig. 2:**
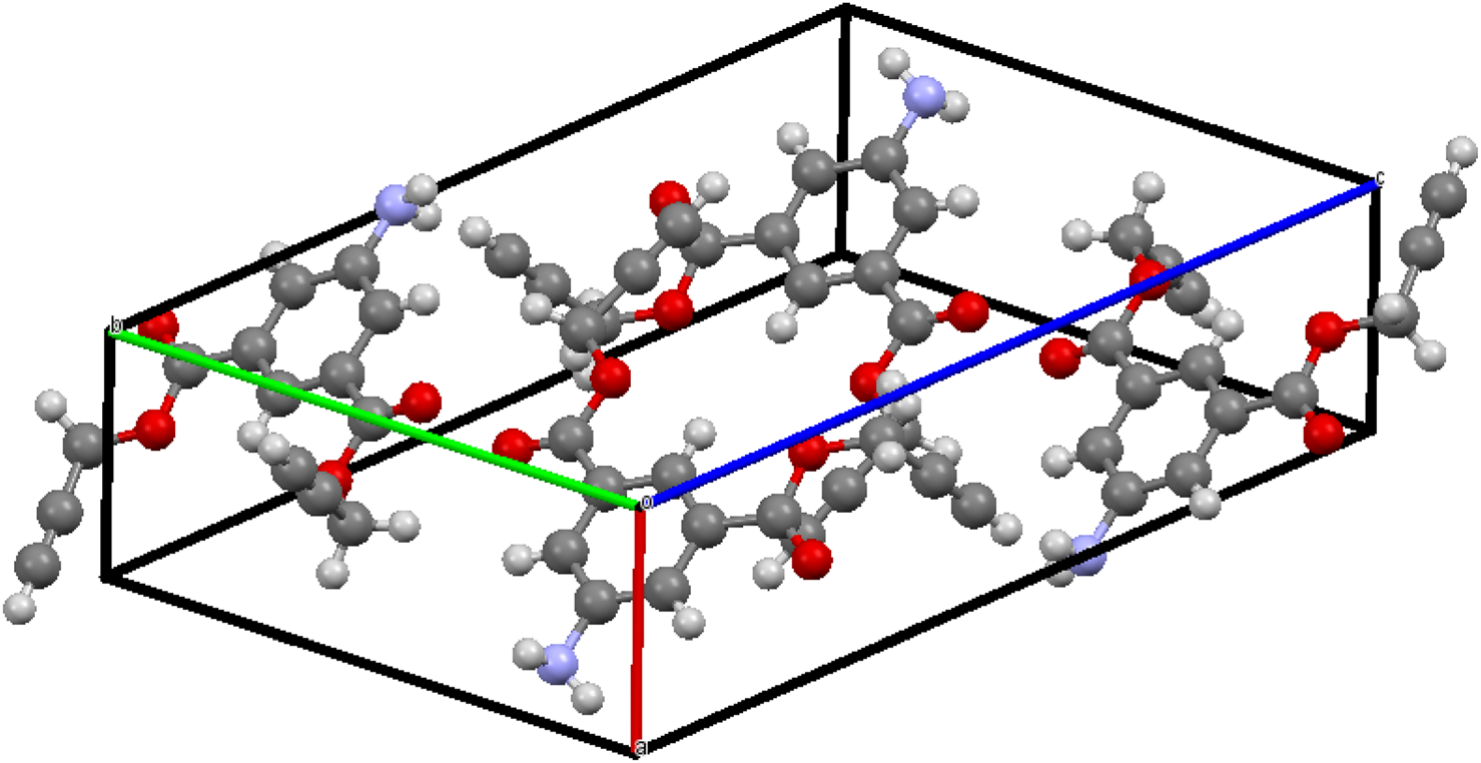
Unit cell (*Z* = 4) showing four molecular units in the asymmetric unit of compound 1.

After undergoing recrystallization with a solution containing acetonitrile and *n*-hexane, the initially formed solid crystalline material successfully transformed into a singular crystal of compound 1, identified by CCDC no. 2300460 and depicted in Fig. 1. The complete crystal data and its structure refinement are presented in Table 1 and Fig. 2.

**Table 1:** Crystallographic data and structure refinement for compound 1

Identification code	1	
Empirical formula	C_13.75_H_10.75_NO_4_	
Formula weight	253.90	
Temperature	216 K	
Wavelength	0.71073 Å	
Crystal system	Monoclinic	
Space group	*P*21/*c*	
Unit cell dimensions	*a* = 4.7711(6)	*α* = 90
	*b* = 13.430(5)	*β* = 94.722(10)
	*c* = 19.544(2)	*γ* = 90
Volume	1248.1(5) Å^3^	
*Z*	4	
Density (calculated)	1.352 g cm^−3^	
Absorption coefficient	0.101 mm^−1^	
*F*(000)	529.0	
Index ranges	*h* = 5, *k* = 11, *l* = 22	
Reflections collected	0.0567(807)	
Completeness to theta = 26.37°	99.7%	
Final *R* indices [*I* > 2 sigma(*I*)]^a^^,^^b^	*R*_1_ = 0.0567, w*R*_2_ = 0.1805	

a *R* = ∑(‖*F*_o_| − |*F*_c_‖)/∑|*F*_o_|.

b *R*_w_ = {∑[*w*(*F*_o_^2^ − *F*_c_^2^)^2^]/∑[*w*(*F*_o_^2^)^2^]}^1/2^.

### 2.3 Synthesis of 2-(azidomethyl)-5-hydroxy-4*H*-pyran-4-one,^28^2

The chloro derivative of kojic acid was synthesized by following the published literature with a few extra tweaks.^29^ In a 50 mL round-bottom flask, 1 mmol of kojic acid was dissolved in 2 equivalents of thionyl chloride at room temperature. The reaction mixture was stirred for 2 h. Upon completion of the reaction, work-up was performed by evaporating the reaction mixture using a rotary evaporator. After drying, the resulting solid was washed with ample water and utilized directly for azidation. A solution of sodium azide (1.5 equivalents) was heated to 85–90 °C for a minimum of 30 min. The obtained chlorokojic solid was then added to the heated solution, and at room temperature, it was stirred overnight. After the reaction, ice-cold water was used to quench the mixture, and the residue was filtered, washed with cold water, and vacuum-dried. The resulting brown solid was recrystallized in THF to obtain a pure compound.

Appearance: brown solid; yield: 85%; mpt: 129–130 °C; FTIR (*ν*_max_ cm^−1^): 3331, 3236, 3059, 3035, 2119, 1658, 1629, 1450, 1379, 1269, 1145, 929, 790, 663, 530; ^1^H NMR (500 MHz, CDCl_3_) *δ* = 8.02 (s, 1H), 7.86 (s, 1H), 6.49 (s, 1H), 4.21 (s, 2H); ^13^C NMR (126 MHz, CDCl_3_) *δ* = 173.74 (C), 162.66 (C), 145.73 (C), 137.81 (CH), 111.18 (CH), 51.13 (CH_2_); HRMS (ESI^+^): [M + H]^+^ cal. for C_6_H_6_N_3_O_3_^+^ is 168.0404, found 168.0413.

### 2.4 General procedure for synthesizing 5-aminoisophthalate-based kojic acid-appended 1,4-disubstituted bis-1,2,3-triazole, 3

First, 12 mL of DMF : H_2_O (8 : 2 v/v) mixture was added to a 100 mL round-bottom flask. The reactants, including 1 mmol of alkyne, 2.1 mmol of organic azide, and CuSO_4_·5H_2_O (10 mol%) along with sodium ascorbate (20 mol%) as a catalyst, were introduced into the flask with slight modifications based on literature procedures. The resulting reaction mixture was stirred at room temperature, and TLC analysis was performed and confirmed the completion of the reaction within 30 min. After the reaction was finished, the mixture was cautiously transferred into a beaker filled with ice-cold water. After filtering, extensively washing with cooled water, and vacuum-drying the resultant solid product, the pure chemical was obtained with an amazing 93% yield.

Appearance: brownish yellow solid; yield: 93%; mpt: 189–190 °C; FTIR (*ν*_max_ cm^−1^): 3442, 3361, 3234, 3140, 3088, 2964, 1722, 1625, 1453, 1350, 1222, 1122, 970, 756; ^1^H NMR (500 MHz, DMSO-*d*_6_) *δ* = 9.32 (s, 2H), 8.36 (s, 2H), 8.06 (s, 2H), 7.61 (s, 1H), 7.38 (s, 2H), 6.43 (s, 2H), 5.62 (s, 4H), 5.39 (s, 4H), 4.94 (s, 2H); ^13^C NMR (126 MHz, DMSO-*d*_6_) *δ* = 173.51 (C), 165.08 (C), 160.35 (C), 149.50 (C), 145.94 (C), 142.11 (C), 139.94 (C), 130.29 (CH), 125.80 (CH), 118.17 (CH), 116.64 (CH), 113.06 (CH), 57.67 (CH_2_), 49.89 (CH_2_); HRMS (ESI^+^): [M + H]^+^ cal. for C_26_H_21_N_7_O_10_^+^ is 592.1423, found 592.1431.

### 2.5 Spectrophotometric analysis

The sensing behavior of probe 3 was thoroughly investigated using UV-visible absorption spectroscopy. Metal ion recognition was explored with a diverse range of metal ions, including Cr^3+^, Mn^2+^, Fe^2+^, Co^2+^, Ni^2+^, Cu^2+^, Zn^2+^, Cd^2+^, Hg^2+^, Pb^2+^, Na^+^, K^+^, Mg^2+^, Ca^2+^, and Ba^2+^. The sensitivity of probe 3 toward Cu^2+^ ions was specifically analyzed using fluorescence spectroscopy. In-depth studies on the time and temperature dependence of the probe, as well as competing metal ion titrations, were conducted to provide a comprehensive understanding of its behavior. The determination of the association constant (*K*_a_) was achieved through the application of a Benesi–Hildebrand (B–H) plot. *K*_a_ was calculated using the formula *K*_a_ = 1/slope, where the slope was obtained by plotting 1/concentration of the analyte *vs.* 1/change in absorbance. Calculations of the limit of detection (LOD) and the limit of quantification (LOQ)^30^ were done using 3*σ*/*S* and 10*σ*/*S*, respectively. In these calculations, *σ* stands for the standard deviation of the blank, and *S* for the slope of the calibration curve. This methodology ensured a rigorous evaluation of the probe's sensing capabilities and provides valuable insights into its potential applications.

### 2.6 Computational studies

#### 2.6.1 Density functional theory (DFT) and Hirshfeld surface analysis

DFT is an effective and adaptable approach to predict a wide range of physiochemical properties in chemical compounds. Its applicability spans various domains, encompassing medicinally significant molecules, molecular probes, metal complexes, and more. DFT serves as an invaluable resource for comprehending and forecasting the diverse properties inherent in these chemical entities.

Utilizing the Gaussian09 program suite,^31^ the stability assessments and geometrical optimizations of 1, 2, and probe 3 were conducted employing the B3LYP^32^/6-311G (d,p) basis set. For complex 3.Cu^2+^, the LanL2DZ basis set^33^ within the DFT/B3LYP method was employed to ascertain the most probable stable molecular configurations. This versatile approach enabled the determination of the molecular structures of the metal complexes, particularly emphasizing their stability. All the optimized geometries were validated by confirming the absence of negative frequencies, ensuring minimum energy on the potential energy surface.

Further analysis involved the calculation of the frontier molecular orbital (FMO) and HOMO–LUMO energy gaps of probe 3 subsequent to its complexation with Cu^2+^ ions, shedding light on the electronic properties. These findings were pivotal in understanding the intricate interplay between the molecular structures and electronic behaviors, offering insights into the reactivity and possible applications of these compounds.

Moreover, Hirshfeld surface (HS) and 2D-fingerprint plots were generated to understand the packing arrangements and intermolecular interactions of the synthesized compounds by using the Crystal Explorer 17.5 program.^34^

#### 2.6.2 Molecular docking

Molecular docking analyses were conducted employing Autodock Vina^35^ and Autodock Tools 1.5.7 to elucidate the protein–ligand interaction processes. The RCSB protein data bank provided pdb format crystal structures of cytochrome P450 14α-sterol demethylase (CYP51) from *Mycobacterium tuberculosis* complexed with fluconazole (PDB ID 1EA1) and *E. coli* topoisomerase II DNA gyrase co-crystallized with clorobiocin (PDB ID 1KZN). Using Gaussview 06, the optimized probe 3 structure was converted to the PDB format. Subsequently, the protein structures were prepared using Auto Dock Tools, which included removing the heteroatoms and inessential H_2_O molecules, introducing polar hydrogens, and adding Kollman charges. The docking pose that exhibited the least binding energy to the receptor was chosen for further analysis. Biovia Discovery Studio Visualizer v21 was utilized for visualization to enhance the interpretability of the results. The outcomes provide insights into the protein–ligand interactions, providing a comprehensive understanding of the docking scenarios.

## 3. Results and discussion

**Scheme 1:**
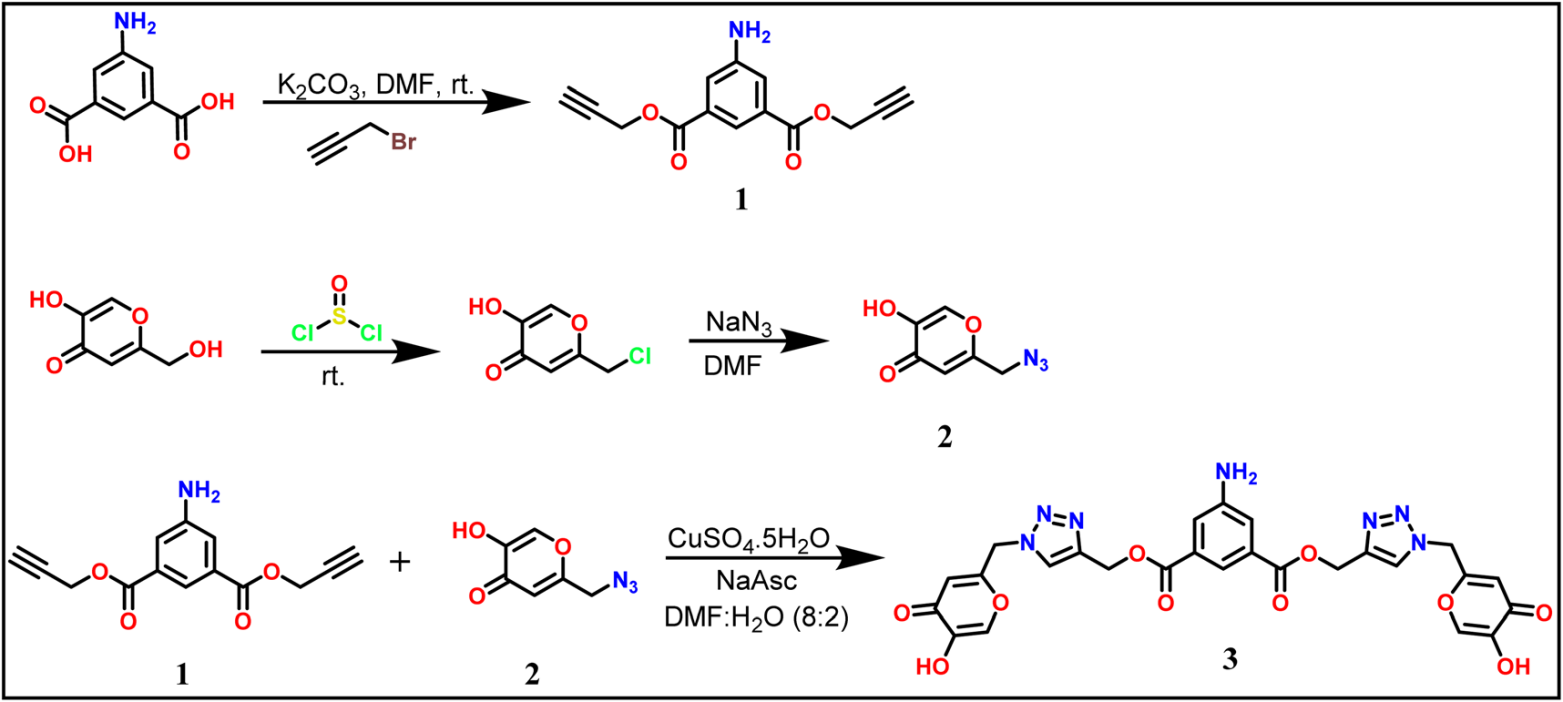
Synthetic route for the 5-aminoisophthalte-linked probe 3.

As mentioned earlier, the terminal bis-alkyne 1 with 5-amino isophthalte was synthesized using the propargylation reaction. The azidation of chlorokojic acid was performed to synthesize the organic azide 2. The proposed probe 3, named bis((1-((5-hydroxy-4-oxo-4*H*-pyran-2-yl)methyl)-1*H*-1,2,3-triazol-4-yl)methyl) 5-aminoisophthalate was synthesized by the reaction between 1 and 2*via* the CuAAC “Click reaction” using CuSO_4_·5H_2_O and NaAsc as a catalyst, as illustrated in Scheme 1. Successful characterizations were obtained for the 5-aminoisophthalte-linked terminal bis-alkyne (1), azide (2), and probe 3 (details are given in the ESI file).†

### 3.1 Spectroscopic analysis

#### 3.1.1 FTIR spectroscopy

The FTIR spectroscopic investigation of 1, 2, and the resulting probe 3 exhibited excellent agreement with the anticipated outcomes. Specifically, alkyne 1 displayed distinctive peaks at 3269 and 2129 cm^−1^, confirming the stretching frequencies of ≡C–H and C≡C bonds, respectively. Likewise, the existence of the azide group (–N_3_) in 2 was affirmed by a sharp peak with high intensity at 2119 cm^−1^ in its spectrum. Upon the addition of 1 and 2, leading to the formation of the 1,2,3-triazole ring, the disappearance of the peaks at 3269, 2129, and 2119 cm^−1^ in the spectrum of 3 indicated the synthesis of a novel compound. Furthermore, a new peak at 3140 cm^−1^ validated the existence of the –C=C–H within the triazole ring.

#### 3.1.2 NMR and mass spectrometry

The successful syntheses of 1 and its 1,2,3-triazole derivative, denoted as 3, were confirmed through ^1^H and ^13^C NMR spectral analysis. In the ^1^H NMR spectrum of alkyne 1, a distinguishing triplet at *δ* = 3.62 ppm was observed, corresponding to the alkynyl protons of 1. Interestingly, this signal was missing in the spectrum of 3. However, the –CH_2_–C– protons of 1 were identified in the proximity of the aromatic triazole moiety in 3. This was initially discerned through the downfield shift of the peak at *δ* = 4.95 ppm in the 1 spectrum. Notably, the peaks at *δ* = 75.35 ppm and *δ* = 77.67 ppm, corresponding to C≡C bonds in the ^13^C NMR spectrum of 1, were conspicuously absent in the spectrum of 3. This absence verified the successful synthesis of probe 3.

The observed molar mass at *m*/*z* = 592.14 further reinforced the confirmation, providing additional support for the synthesis of the desired compound.

### 3.2 UV-Vis analysis

**Fig. 3:**
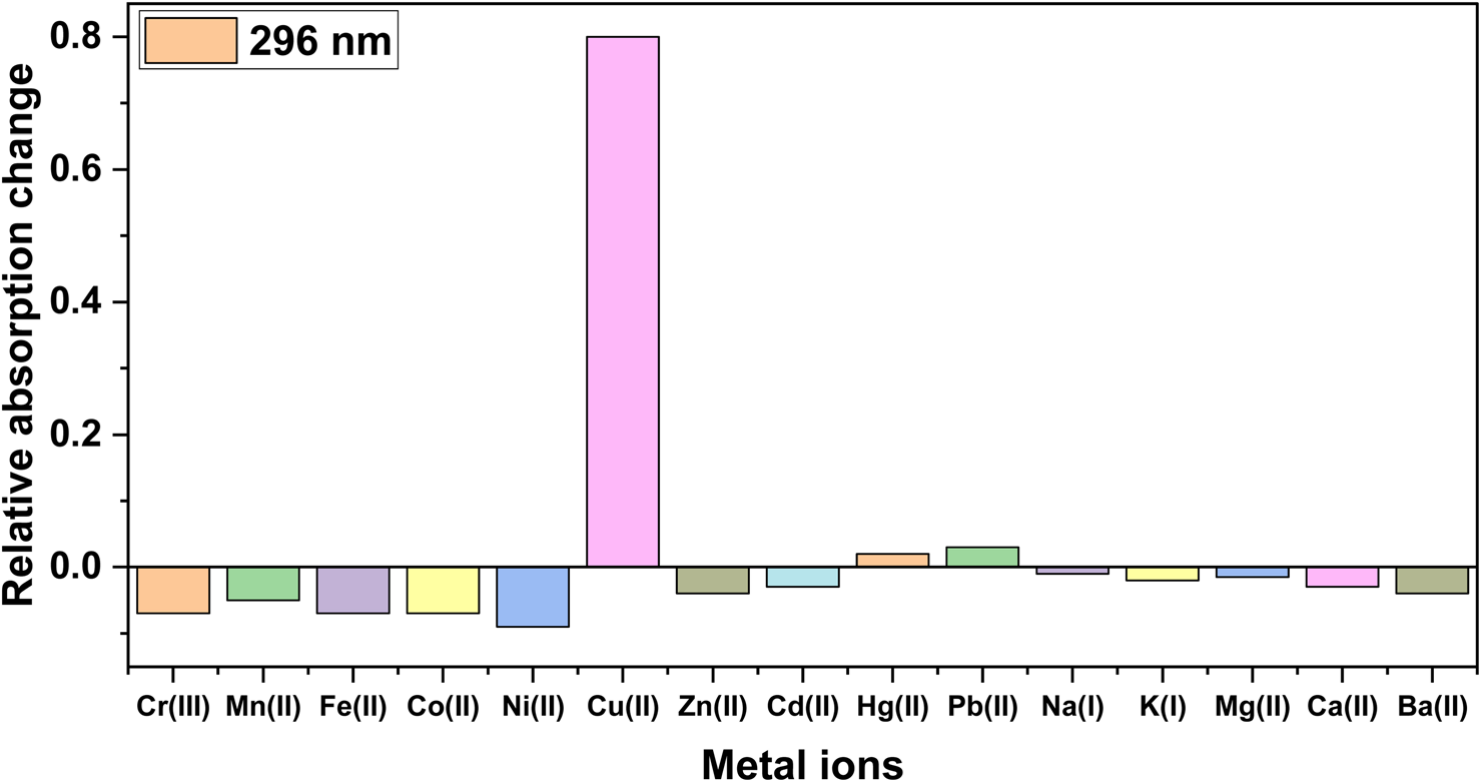
Relative absorption intensity changes of probe 3 on adding different metal ions in DMSO/H_2_O (4 : 1).

The expected fine-quality UV-Vis absorption characteristics of the synthesized 5-aminoisophthalate-based kojic acid-appended 1,4-disubstituted bis-1,2,3-triazole as probe 3 were attributed to its rich aromatic and conjugated moieties embedded within its molecular structure. Subsequent absorbance analysis confirmed these expectations, revealing that probe 3 displayed a prominent absorption peak at *λ*_max_ = 296 nm. Following this, the potential of the probe for metal ion recognition was explored, wherein 0.5 mM solutions of both probe 3 and various metal ions, including Cr^3+^, Mn^2+^, Fe^2+^, Co^2+^, Ni^2+^, Cu^2+^, Zn^2+^, Cd^2+^, Hg^2+^, Pb^2+^, Na^+^, K^+^, Mg^2+^, Ca^2+^, and Ba^2+^ were employed. The UV-Vis analyses to assess the probe's ion recognition capabilities were carried out in a solvent medium consisting of a 4 : 1 ratio of DMSO/H_2_O. The absorption of the chemosensor remained largely unaffected by the majority of the tested metal ions. Notably, only Cu^2+^ ions induced a noticeable shift in absorption, thereby establishing the sensor's selective responsiveness to Cu^2+^ ions, as depicted in Fig. 3, where the relative absorption change of the probe for different metal ions is presented. The relative absorption change was determined by subtracting the absorbance of the reference solution from the absorbance of the solution containing the metal ion, which gave the change in absorbance due to the presence of that specific metal ion and could be then expressed as the relative change compared to the reference absorbance.

### 3.3 Chemosensing potential of probe 3 for Cu^2+^

**Fig. 4:**
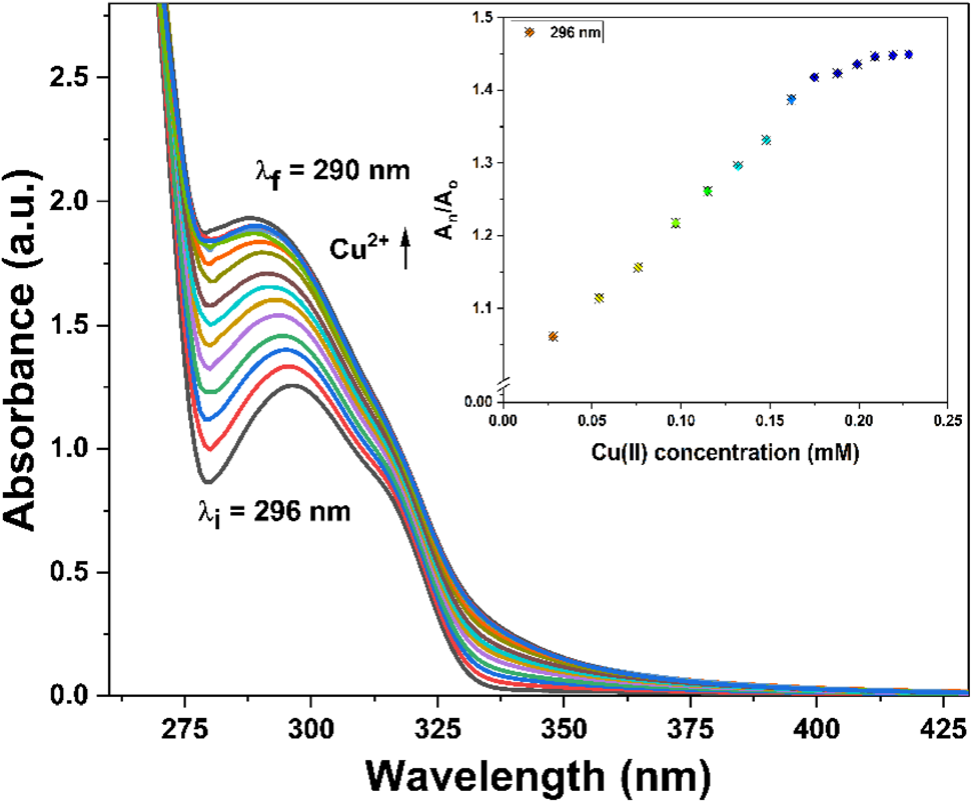
Systematic titration of probe 3 with the gradual addition of Cu^2+^ ions; the inset represents the correlation between the probe's relative absorption intensity (*A*_n_/*A*_o_) and the Cu^2+^ concentration (mM).

The selective recognition behavior of probe 3 for Cu^2+^ ions was investigated through UV-Vis spectroscopic analysis, wherein a 0.5 mM solution of the probe in a 4 : 1 mixture of DMSO and H_2_O was sequentially subjected to gradual additions of Cu^2+^ ions (0–0.25 mM). The absorbance of the solution was measured after each successive addition, enabling the assessment of the probe's recognition potential. Fig. 4 illustrates the alterations in the absorption spectra of probe 3 as Cu^2+^ ions were progressively introduced. Initially, probe 3 exhibited a well-defined absorption peak at *λ*_max_ = 296 nm. However, with the introduction of the metal ions, there was a gradual hyperchromic response, accompanied by a shift toward a shorter wavelength (blue shift) of approximately 6 nm, ultimately leading to a shift in *λ*_max_ to 290 nm upon completion of the titration. Furthermore, *K*_a_ for the interaction of Cu^2+^ ions with 3 determined through the B–H eqn (1) was 4.89 × 10^3^ M^−1^, and the B–H plot for the same is illustrated in Fig. SI-14.†1
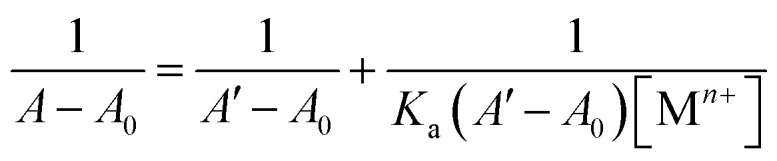
where *A*_0_ is the initial absorbance intensity, *A* is the absorbance intensity with a particular concentration of metal ions, *A*′ is the final absorbance intensity, [M^*n*+^] is the concentration of metal ions, and *K*_a_ is the association constant.

### 3.4 Competitive metal ion titration analysis

**Fig. 5:**
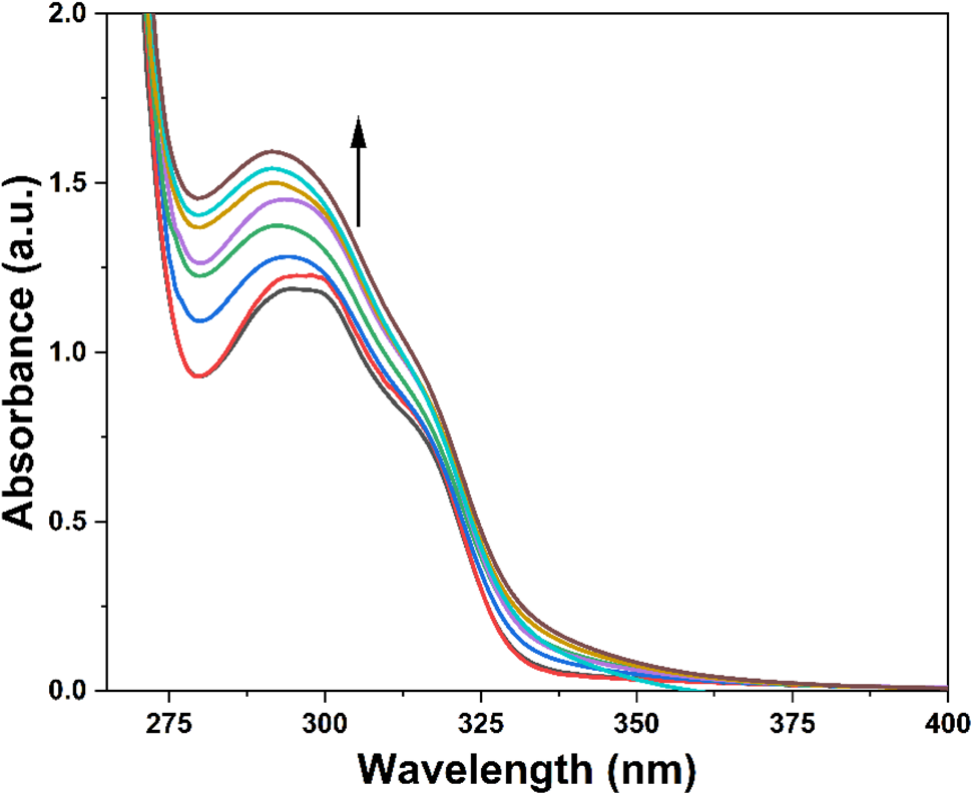
Absorption spectra of probe 3 indicating the selective recognition of Cu^2+^ ions among different metal ions in equimolar concentrations.

The competitive analysis of metal ions holds a crucial role in analytical chemistry, particularly in assessing the practical utility of 1,2,3-triazole-based chemosensors in addressing environmental concerns and optimizing industrial processes. To evaluate the specificity of probe 3, a competitive metal ion titration was conducted in a solvent system consisting of DMSO/H_2_O (4 : 1 v/v) using an equimolar mixture of various metal ions (Fig. 5). The absorption spectra observed after the titration suggested that the probe could selectively detect Cu^2+^ ions, regardless of the presence of other ions. This was evident from the resemblance of the recorded absorption spectra to those obtained when probe 3 was subjected to Cu^2+^ ions only.

### 3.5 Time and temperature-dependent analysis

The assessment of a chemosensor's sensitivity and stability in selectively binding metal ions requires a thorough examination of its response to changes in time and temperature. Analyzing the influence of these variables on the sensor's performance is essential for optimizing its sensitivity and selectivity, ensuring a precise and dependable detection of target analytes. In pursuit of this objective, an experiment was conducted to investigate how time and temperature impact the binding affinity of probe 3 for Cu^2+^ ions. The results are presented in Fig. SI-15† regarding the effect of time on the binding of Cu^2+^ ions with probe 3. It was observed that the absorption intensity of the probe 3.Cu^2+^ complex solutions remained constant even as the interaction duration was extended. This observation indicated that the probe's binding affinity for Cu^2+^ ions was time-independent.

In the context of temperature-dependent analysis, probe 3.Cu^2+^ solution was subjected to a temperature range from 20 °C to 50 °C, and the absorption spectra were collected at 2 °C intervals. Fig. SI-16† illustrate the temperature effects on Cu^2+^ binding to probe 3, revealing that variations within this temperature range had no significant impact on the stability of probe 3.Cu^2+^ complexation. Thus, the complexation was found to be stable across the examined temperature range.

### 3.6 Reversibility analysis

The reversibility of a chemosensor is a crucial aspect in the field of analytical chemistry, providing insights into the dynamic interactions between the chemosensor and the target analyte. The significance of this analysis lies in the ability to discern the reversibility of the chemosensor-analyte complex formation, which has implications for its practical utility in various sensing applications. In this context, the 3.Cu^2+^ complex solution was subjected to the incremental addition of ethylenediaminetetraacetic acid (EDTA), which resulted in a decrease in absorbance (Fig. SI-17†), attributable to a competitive binding scenario, wherein EDTA competed with the chemosensor for Cu^2+^ binding sites. This competition was due to the higher affinity of EDTA for Cu^2+^ ions, leading to the displacement of the chemosensor and subsequent alteration in the optical properties of the solution. However, these preliminary results indicated that probe 3 could be used as both a reusable sensor and as a scavenger of copper ions depending on the concentration of both Cu^2+^ and EDTA, which may help address the toxicity concerns of Cu^2+^ ions.

### 3.7 Fluorescence investigation

**Fig. 6:**
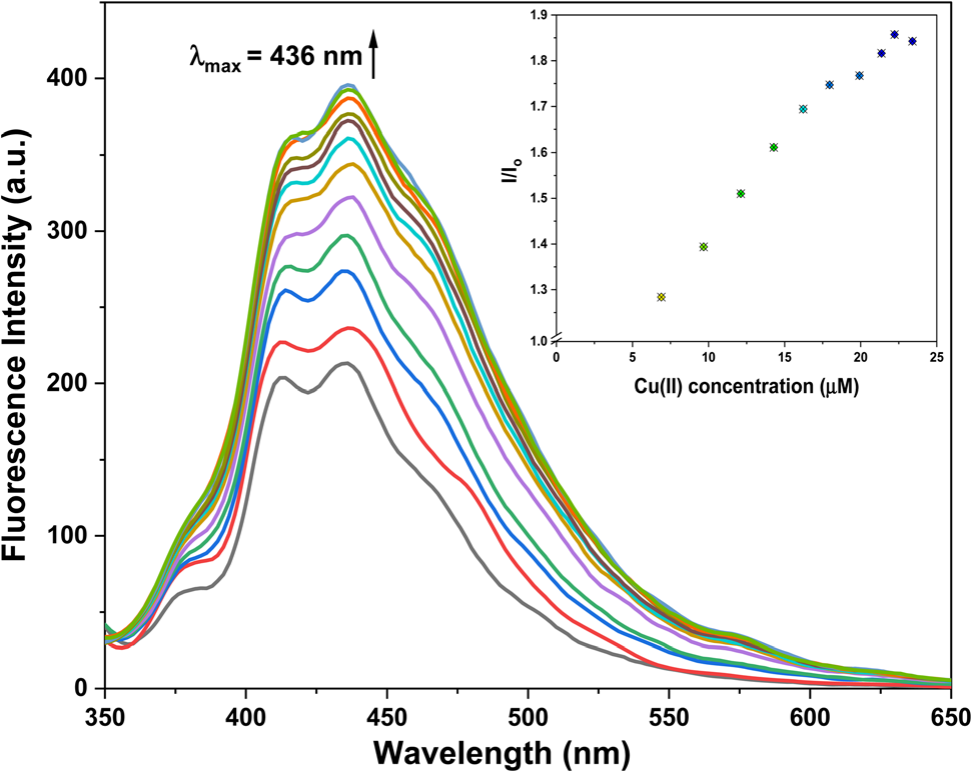
Fluorescence titration of probe 3 with the successive addition of Cu^2+^ ions; inset exhibits the relative emission of 3 (*I*/*I*_o_) as a function of Cu^2+^ concentration (μM).

The photophysical attributes of probe 3 were examined through fluorescence spectroscopy analysis in conjunction with UV-Vis absorption spectroscopy analysis. The emission of a 10 μM probe 3 solution excited at 340 nm (*λ*_ex_) was monitored, revealing a prominent emission (*λ*_em_) at approximately 436 nm accompanied by a shoulder peak at 414 nm (*ϕ* = 0.27). Upon the successive addition of Cu^2+^ ions (0–25 μM), a significant enhancement of the fluorescence emission of the probe was observed, as depicted in Fig. 6, exhibiting almost a two-fold enhancement in the fluorescence intensity (*ϕ* = 0.96), which confirmed the recognition of the Cu^2+^ ions by 3. The LOD and LOQ were calculated by analyzing the correlation plot (Fig. SI-18†) derived from the fluorescence titrations and determined to be 8.82 μM and 29.39 μM, respectively.

Moreover, Table 2 compares the LODs between previously synthesized chemosensors and the present study, employing 1,4-disubstituted 1,2,3-triazoles for Cu^2+^ ion detection. The LOD assumes particular significance in environmental monitoring, especially concerning the metal ion concentration limits established by the Environmental Protection Agency (EPA). It signifies the minimum concentration at which a specific metal ion can be consistently identified and quantified, confirming the precision and accuracy of analytical methodologies. Adhering to or exceeding the EPA's stipulated detection limit is imperative for protecting public health and the environment. This compliance allows for recognizing and regulating potentially hazardous metal impurities in air, water, and soil, thereby facilitating the implementation of operative pollution control and risk management strategies.^36^

**Table 2:** Comparison of LODs from previously developed chemosensors for Cu^2+^ ions^a^

S. no.	Chemosensor	Structure	Limit of detection	Reference
1	CBT	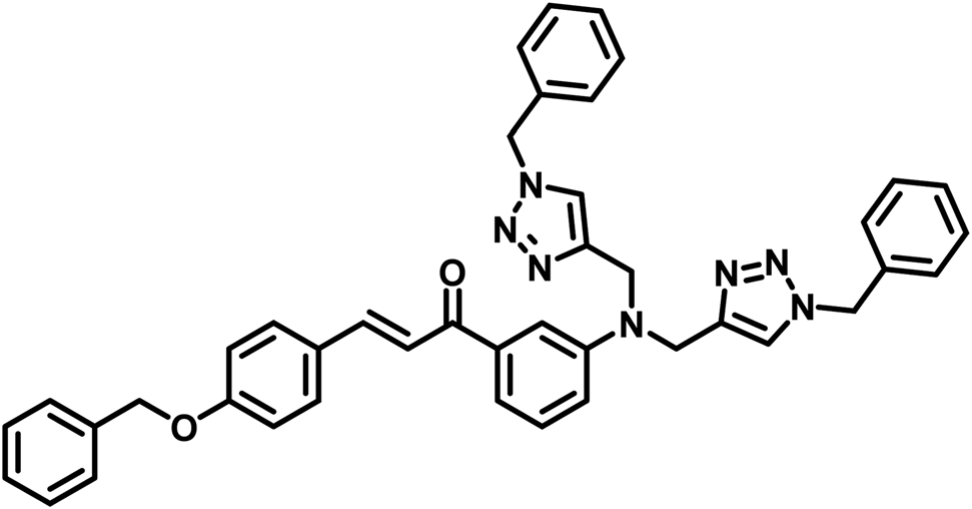	110 μM	37
2	APT1	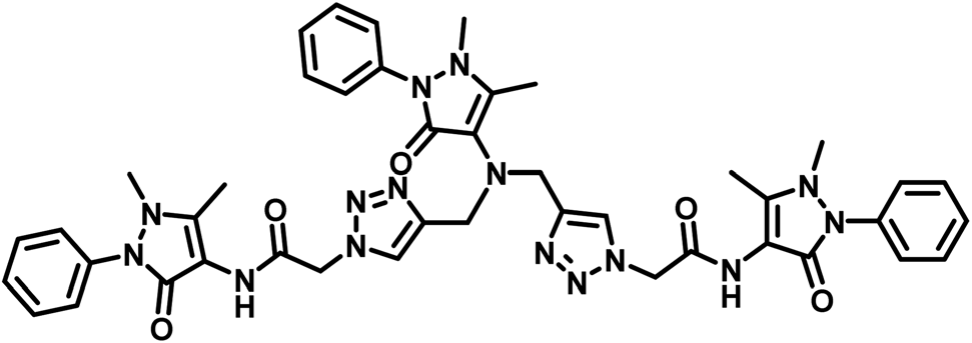	63 μM	30
3	APT2	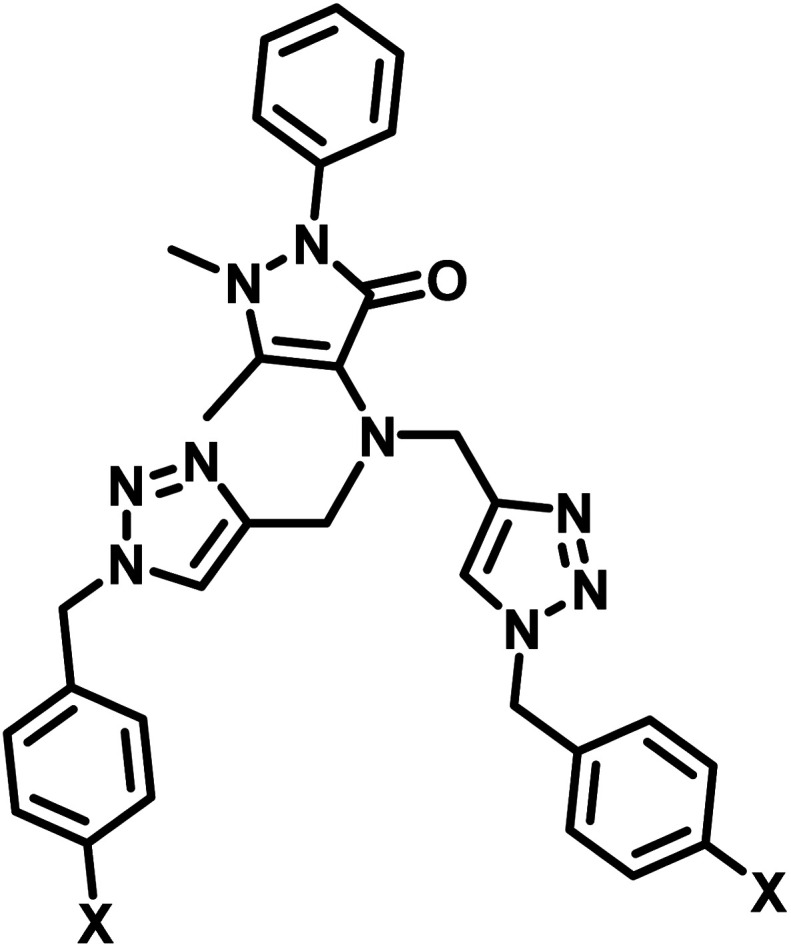 (a) X = Cl	0.14 mM	11
		(b) X = CN	0.20 mM	
4	CFT	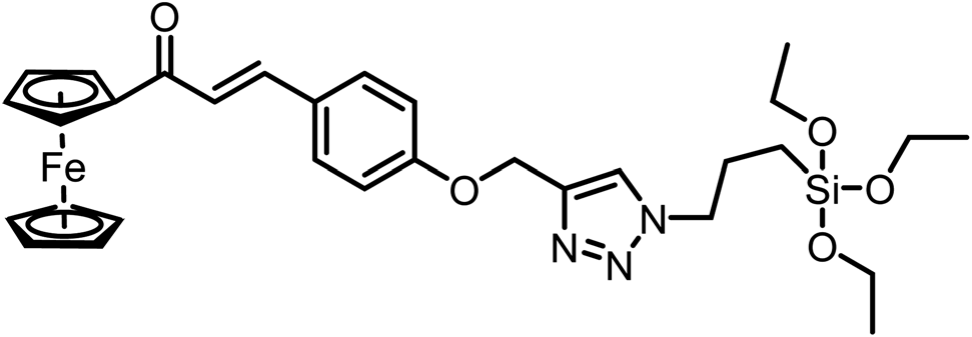	13.4 μM	38
		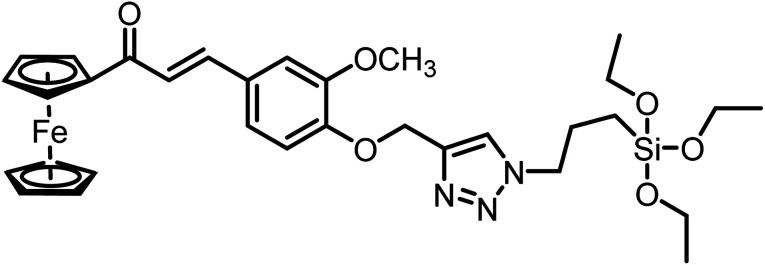	9.42 μM	
5	Carbohydrate based sensors	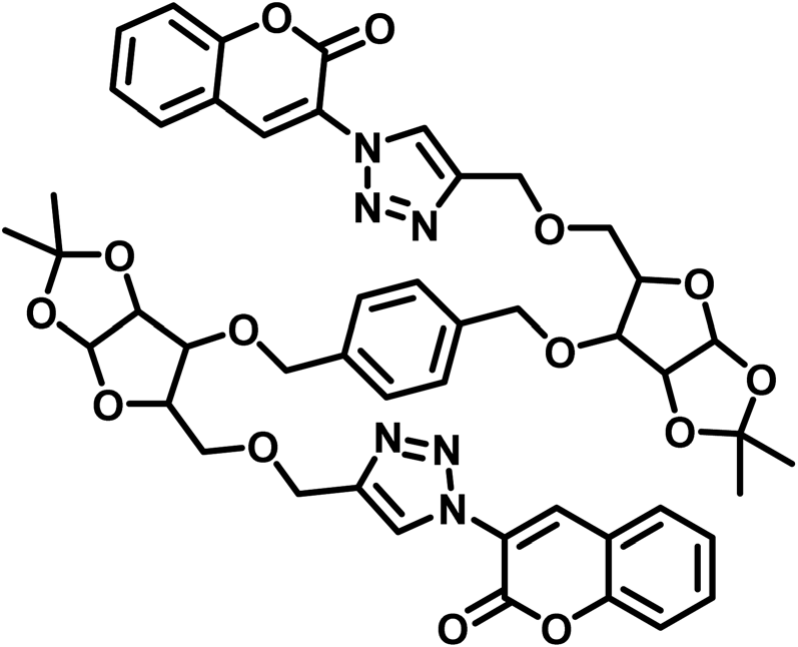	6.99 μM	39
		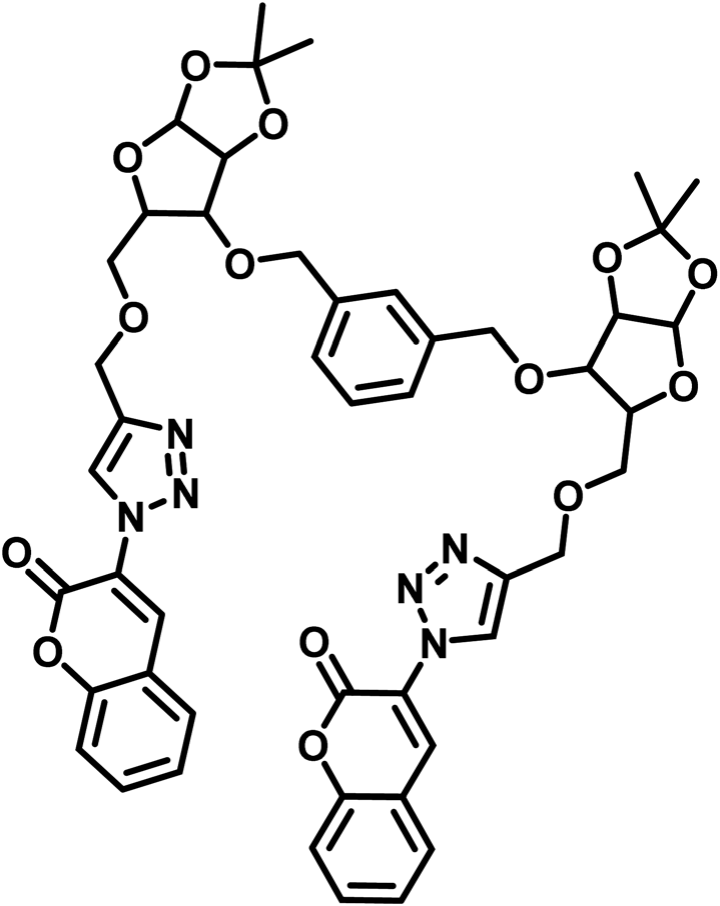	7.30 μM	
6	Probe 3	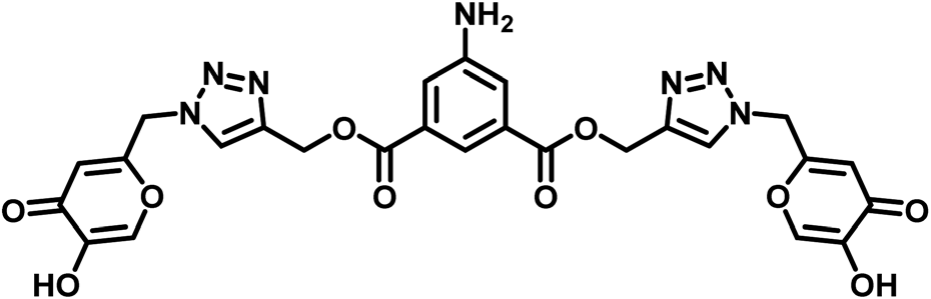	8.82 μM	This work

a CBT = chalcone-based 1,2,3-triazole; APT1 = antipyrine based bis-1,2,3-triazole; APT2 = antipyrine linked bis-1,2,3-triazole, CFT = ferrocene–chalcone–triazole.

### 3.8 Job plot analysis

**Fig. 7:**
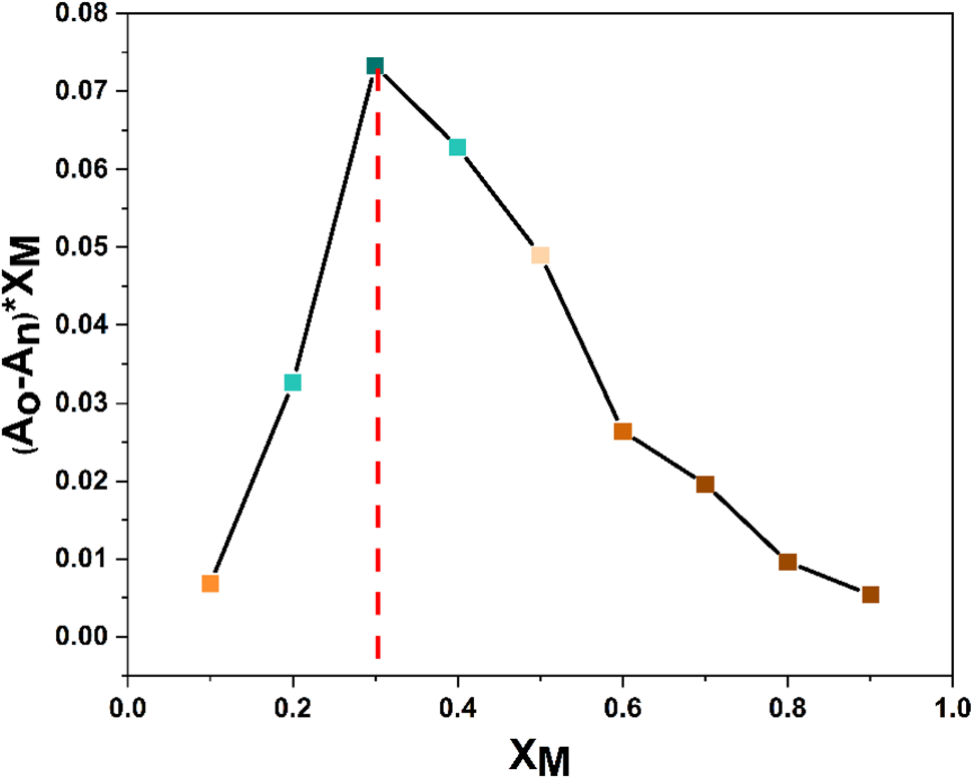
Job plot analysis of probe 3 upon Cu^2+^ complexation indicating a 2 : 1 (L : M) stoichiometry.

In order to ascertain the stoichiometry between probe 3 and Cu^2+^ ions, a Job's plot analysis was conducted, which involved preparing a range of solutions of probe 3 and Cu^2+^ ions with equal concentrations while systematically altering the molar ratio of the probe (or metal ion) from 0.1 to 0.9. The absorbance of each of these solutions was measured, and the plot of the relative absorption change *versus* the molar ratio of the metal ions was analyzed. The positions of the maximum absorbance peaks in the graph represent the ratio of ligand to metal in the complex solution. For instance, a maximum absorbance corresponding to a mole fraction ratio of 0.5 on the metal mole ratio scale suggests a complex with a 1 : 1 ligand-to-metal (L : M) composition. Similarly, peaks at 0.33 and 0.67 indicate complexes with L : M ratios of 2 : 1 and 1 : 2, respectively. As illustrated in Fig. 7, the maximum absorbance was at the 0.3 molar ratio, indicating a stoichiometric ratio of 2 : 1 (L : M) between the probe and the metal ions.

### 3.9 Computational studies

#### 3.9.1 Structure optimization and FMO analysis

**Fig. 8:**
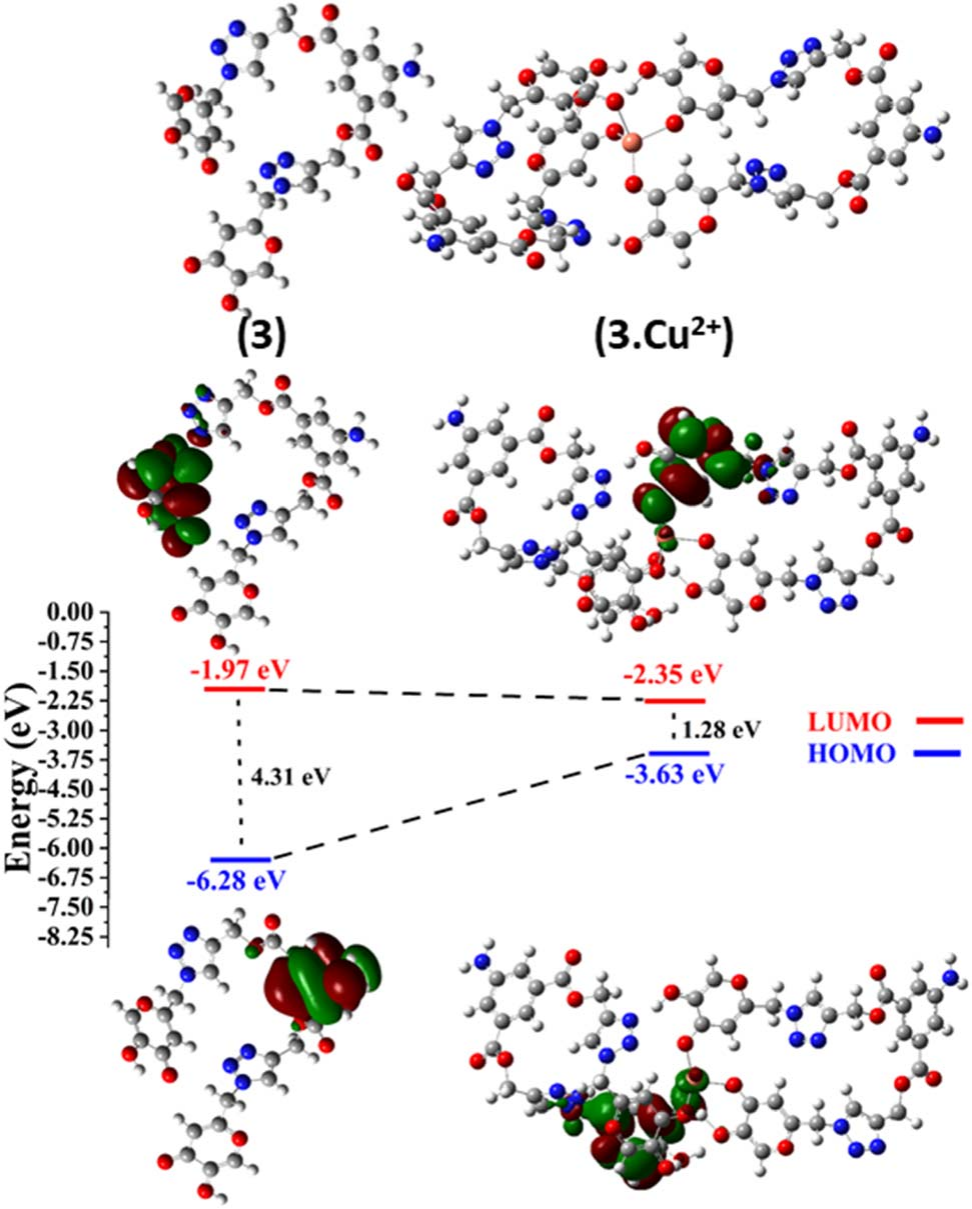
Optimized geometries of probe 3 and complex 3.Cu^2+^ and their contour plots.

The structures of 1, 2, and probe 3 were optimized through DFT calculations using the B3LYP/6-311G (d,p) basis set. In the case of complex 3.Cu^2+^, the DFT/B3LYP method was applied with the LanL2DZ basis set to elucidate the most likely stable molecular configurations. FMOs play a crucial role in defining various molecular properties, including reactivity, light absorption, and electrical properties. Notably, the HOMO serves as an electron donor, while the LUMO functions as an electron acceptor. The difference between the HOMO and LUMO FMOs serves as a determinant of a compound's chemical stability. Optimized structures of 1, 2, and probe 3 and their contour plots are shown in Fig. SI-19.† Meanwhile, Fig. 8 provides a graphical representation of the FMOs and their corresponding energy gaps for probe 3 and complex 3.Cu^2+^. In probe 3, the electron density of the HOMO is localized on the 5-aminoisophthale group, while the LUMO is localized on the kojic acid of one arm of bis-1,2,3-triazole. In contrast, in 3.Cu^2+^, the attachment of the Cu^2+^ ions modifies the FMO distribution. In both the HOMO and LUMO, the electron density resides in between the two ligands having a Cu^2+^ ion attached to them. The depicted information shows that the energy gap of complex 3.Cu^2+^ was less than that of probe 3, indicating the development of an extra stable bond between probe 3 and the Cu^2+^ metal ion.

#### 3.9.2 Hirshfeld surface, 2D-fingerprint plot and 3D-interaction energy frameworks analysis

In crystal engineering, the intermolecular interactions of the title compound are analyzed using Hirshfeld surface (HS) analysis *via* Crystal Explorer^34^ version 17.5. This computational technique allows for the visualization and quantification of interactions within crystalline materials. By importing a CIF file into Crystal Explorer 17.5, three-dimensional Hirshfeld surfaces (3D-HS) and their corresponding two-dimensional fingerprint plots can be generated. The HS method partitions the space within a crystal based on the ratio of promolecule to procrystal electron densities, typically at a value of 0.5. This surface is then mapped using the normalized contact distance (*d*_norm_), which is calculated in terms of external and internal distances (*d*_e_ and *d*_i_, respectively) as well as the van der Waals (vdW) radii of atoms. The *d*_norm_ values can be negative or positive, indicating whether intermolecular contacts are shorter or longer than the respective vdW separations. Specifically, contacts with distances equal to the sum of the vdW radii are depicted in white, while those with distances shorter or longer than the vdW radii are represented in red and blue, respectively.^40^ This analysis provides insights into various types of intermolecular interactions, including H⋯H, C⋯H, O⋯H, and H⋯O contacts within the crystal lattice.

**Fig. 9:**
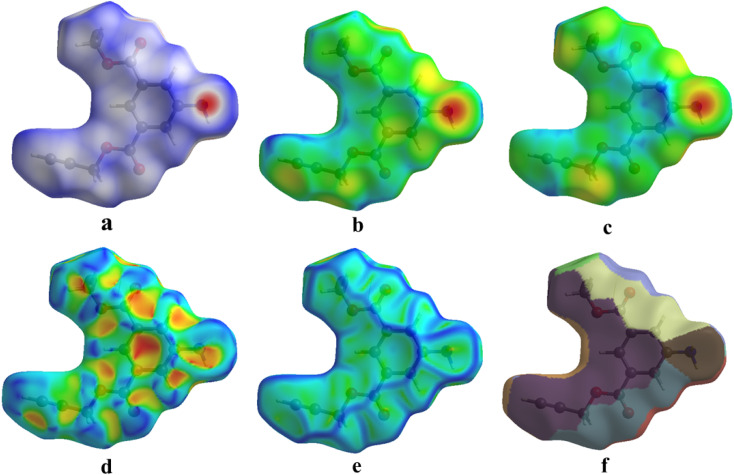
(a) *d*_norm_, (b) *d*_i_, (c) *d*_e_, (d) shape index, (e) curvedness, and (f) fragment patches with a selected network of interactions of title compound 1.

Whenever the distance between atoms equals the total of their van der Waals radii, a white spot appears on the HS. Each point on the HS signifies two distances: (i) the distance from the nearest outer nucleus to the surface (denoted as *d*_e_), and (ii) the distance from the surface to the closest inner nucleus (referred to as *d*_i_). The HS is shown to be a transparent surface through which the structure of the molecules can be seen. The HS plots over *d*_norm_, *d*_i_, *d*_e_, shape index, curvedness, and fragment patches with a selected network of interactions for the title compound 1 are shown in Fig. 9.

**Fig. 10:**
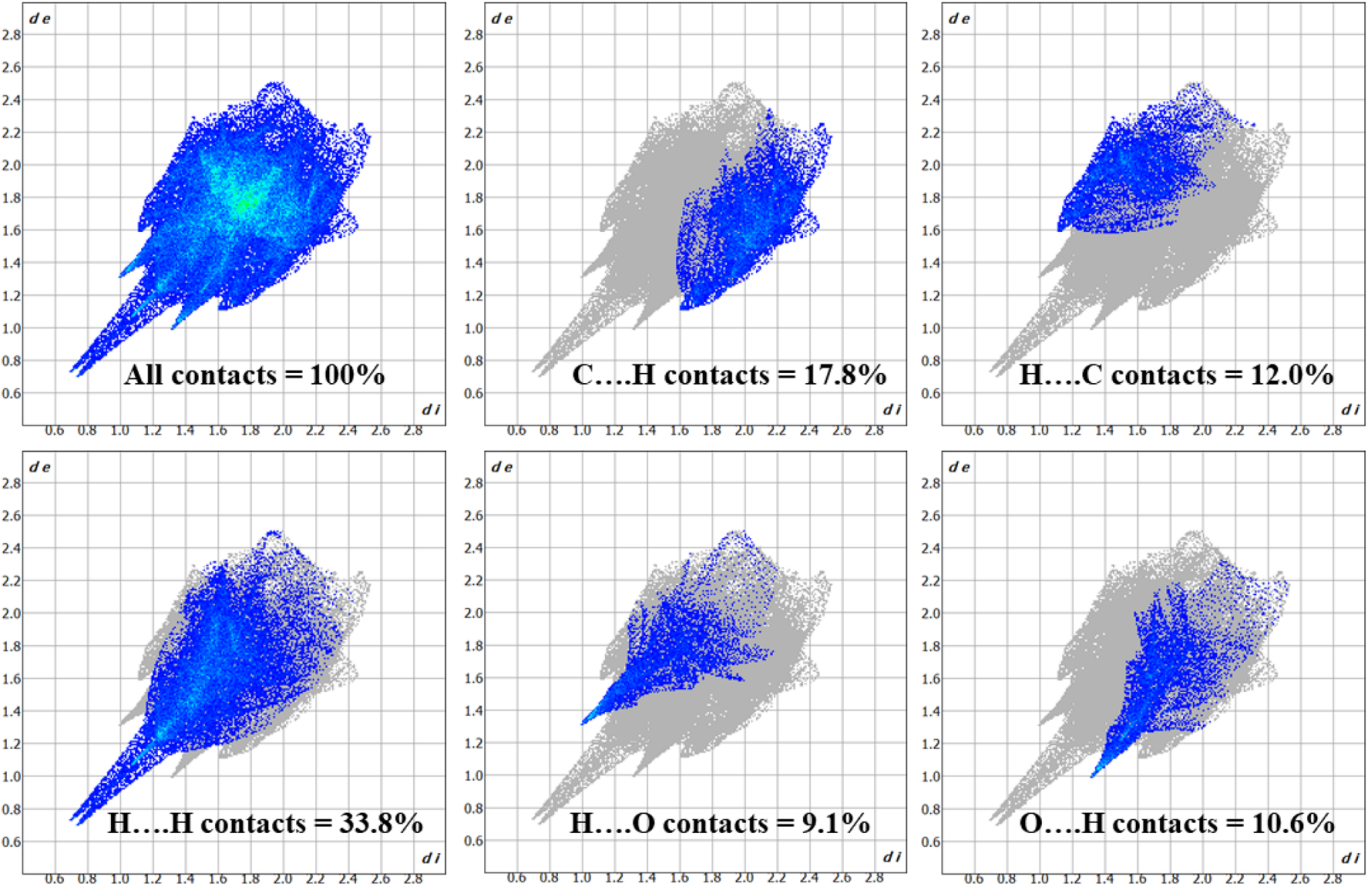
2D fingerprint plots for title compound 1, showing contributions from different contacts.

The 3D *d*_norm_ surface can be resolved into 2D fingerprint plots, which analyze all intermolecular contacts at the same time and give a quantitative summary of the nature and type of intermolecular contacts experienced by the molecules in a crystal. Furthermore, we may get a concise understanding of the relative% contribution and individual contact separation from various contact types in the entire HS area by 2D-fingerprint plots (*d*_e_*vs. d*_i_).^41^ The 2D fingerprint plot provides a detailed two-dimensional graphical illustration of the intermolecular contacts in a crystal system. Fig. 10 represents the 2D fingerprint plots for the title compound 1, which illustrate distinct intermolecular interactions concerning the HS area. H⋯H was the most significant contact in the crystal packing of 1, accounting for 33.8% of the total, while contacts such as C⋯H (17.8%), H⋯C (12.0%), H⋯O (9.1%), and O⋯H (10.6%) provided only a small contribution to the crystal packing.

Crystal Explorer 17.5 program was used to perform the energy framework calculations utilizing symmetry operations on the B3LYP/6-311G(d, p) functional basis set.

A collection of atoms with a radius of 3.8 Å was formed around a molecule. The computation process next involved figuring out the various kinds of molecular interaction energies.^42^ All the electrostatic energy (*E*_ele_) is related to the forces that exist between charged particles, whereas polarization energy (*E*_pol_) is focused on the distortion of an electron cloud inside a molecule as a result of other nearby charge distributions. The term ‘dispersive energy, *E*_dis_’ describes the weak attractive forces resulting from momentary variations in the electron distribution of a molecule. The amount of energy required to overcome the forces preventing two molecules from occupying the same space is known as repulsive energy (*E*_rep_). The overall interaction energy of the molecule under investigation may be obtained by summing the various kinds of interaction energies, as the formula below illustrates:

2 *E*_tot_ = *E*_pol_ + *E*_ele_ + *E*_dis_ + *E*_rep_

**Fig. 11:**
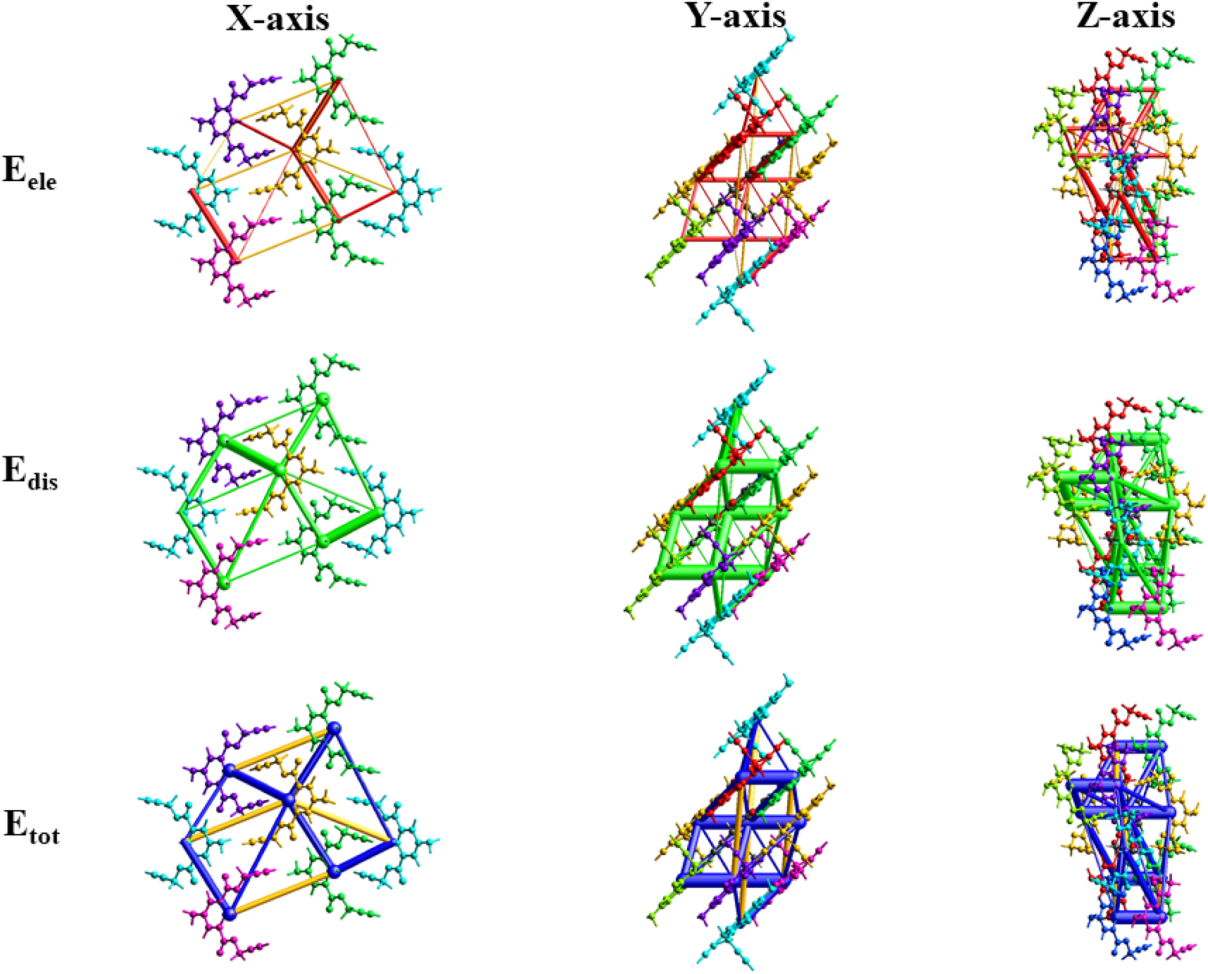
3D-interaction energy frameworks for compound 1 within the radius of 3.8 Å along the *x*, *y*, and *z* axes.

Fig. 11 illustrates the molecule used in the estimation of the interaction energies along the *x*, *y*, and *z* axes for compound 1. All types of interaction energies between the molecular pairs were calculated and are listed in Table 3 for compound 1. Herein, *R* shows the distance among the mean atomic spots (molecular centroids) and *N* signifies the no. of molecules at a specific distance. The scaling factors that were employed for all types of interaction energies for the B3LYP/6-311G(d, p) basis set were *k*_ele_ = 1.057, *k*_pol_ = 0.740, *k*_disp_ = 0.871, and *k*_rep_ = 0.618,^43^ and energy values for each type of interaction are reported in kJ mol^−1^. A molecule exhibiting *x*, −*y* + 1/2, and *z* + 1/2 symmetry operations, positioned at a distance of 10.53 Å from the chosen molecule's centroid, demonstrates a maximum total interaction energy of 19.0 kJ mol^−1^, as Table 3 illustrates. On the other hand, the symmetry operation *x*, *y*, and *z* situated at 4.77 Å from the chosen molecule's centroid yields the lowest total interaction energy of −46.3 kJ mol^−1^. Fig. 11 depicts the *E*_ele_, *E*_dis_, and *E*_tot_ for compound 1 along the *x*, *y*, and *z* axes, depicted in red, green, and blue, respectively.

**Table 3:** Interaction energies (kJ mol^−1^) for a unit cell of the crystal structure of compound 1^a^

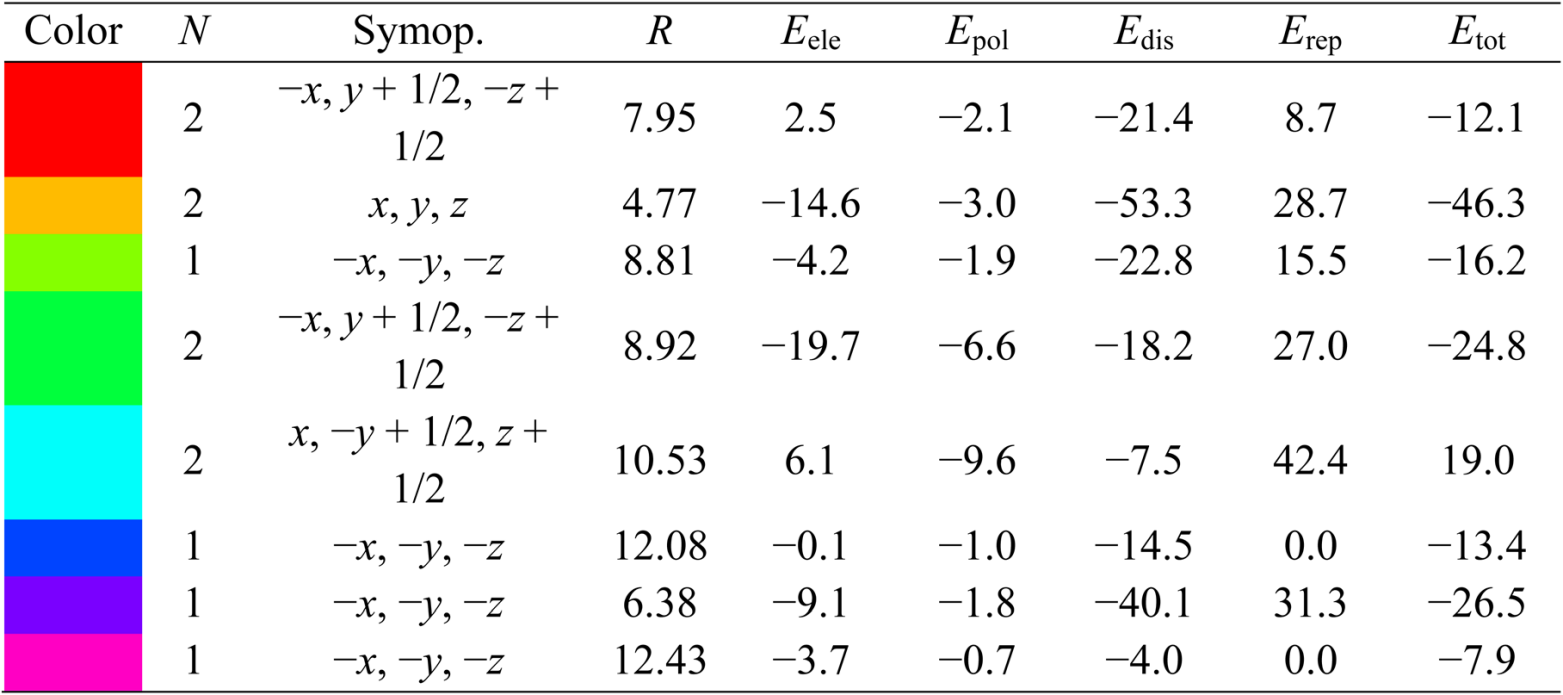

a *R* is the distance between molecular centroids (mean atomic position) in Å and *N* is the number of molecules at that distance, Symop = rotational symmetry operator, *E*_ele_ = electrostatic energy, *E*_pol_ = polarization energy, *E*_dis_ = dispersion energy, *E*_rep_ = repulsion energy, *E*_tot_ = total interaction energy, energies are in kJ mol^−1^.

#### 3.9.3 Molecular docking

**Fig. 12:**
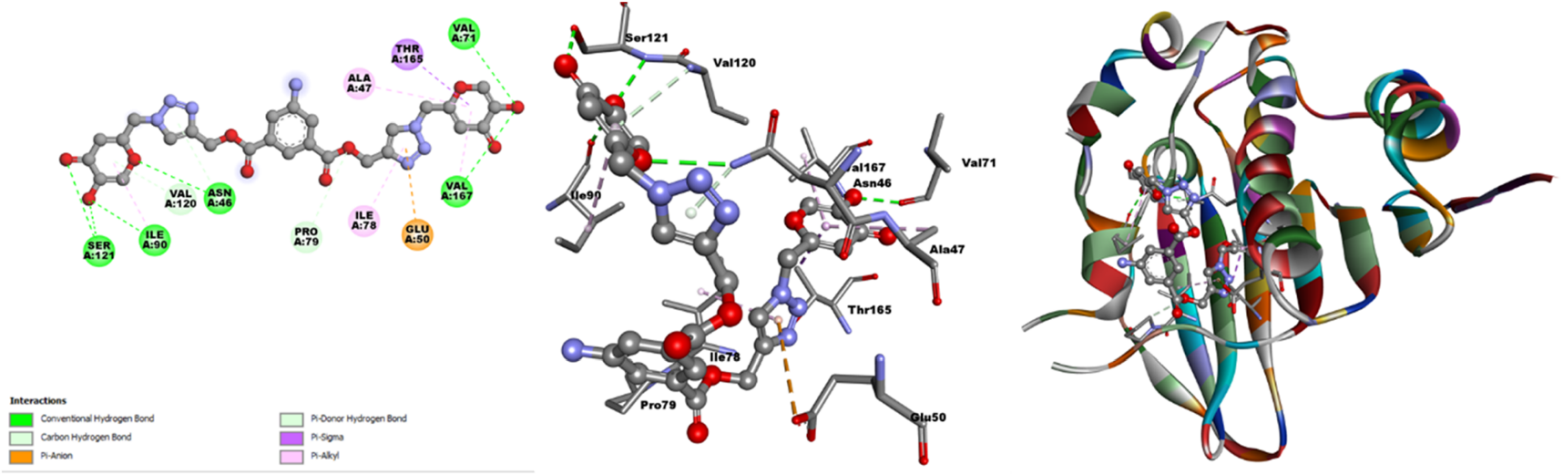
2D and 3D interactions and an illustration of probe 3 docked in the active site of DNA gyrase topoisomerase II.

The conformation of molecular probe 3 underwent optimization through molecular docking simulations to identify its energetically favorable docked structures. Examining the results in Table SI-2† revealed a prominent docking score of −9.2 kcal mol^−1^ for probe 3 when interacting with the bacterial enzyme *E. coli* DNA gyrase topoisomerase II (PDB ID 1KZN). Within this interaction, six distinct hydrogen bonds were observed between SER121, ILE90, ASN46, VAL71, and VAL167, acting as hydrogen donors to the oxygen atom of the kojic acid group, denoted by dotted green lines (Fig. 12). Moreover, the π-electron density of the 5-aminoisophthalate moiety displayed an interaction with PRO79, while the π-orbitals of the benzene and triazole ring established π-alkyl interactions with ALA47 and ILE78.

Similarly, when engaged with the fungal enzyme 14α-demethylase lanosterol (PDB ID 1EA1), probe 3 exhibited a docking score of −8.8 kcal mol^−1^, as illustrated in Table SI-3.† In particular, interactions involving six hydrogen bonds between ASP67, GLN72, PHE255, THR260, and ALA256 were identified, serving as hydrogen donors to the oxygen atom of the kojic acid group. Additionally, TYR76 acted as a hydrogen donor to the nitrogen atom of the 5-aminoisophthalate group. The benzene and triazole ring system of probe 3 showed π-orbital interactions with LEU321, ARG95, and MET79 (Fig. SI-20†). Alongside these interactions, various π-interactions, including π–π T-shaped, π–anion, and π–sigma, were observed. The smaller bond length and the more negative docking score signify enhanced the stability of the hybrid molecules. Molecular docking analyses suggested promising results, primarily for the bacterial activities. The representation of the potent molecular probe 3 docked within the active site of DNA gyrase topoisomerase II (PDB ID 1KZN) is depicted in Fig. 12. The strategic molecular docking approach aimed to augment the antimicrobial efficacy of probe 3.

### 3.10 Probable binding mode

**Fig. 13:**
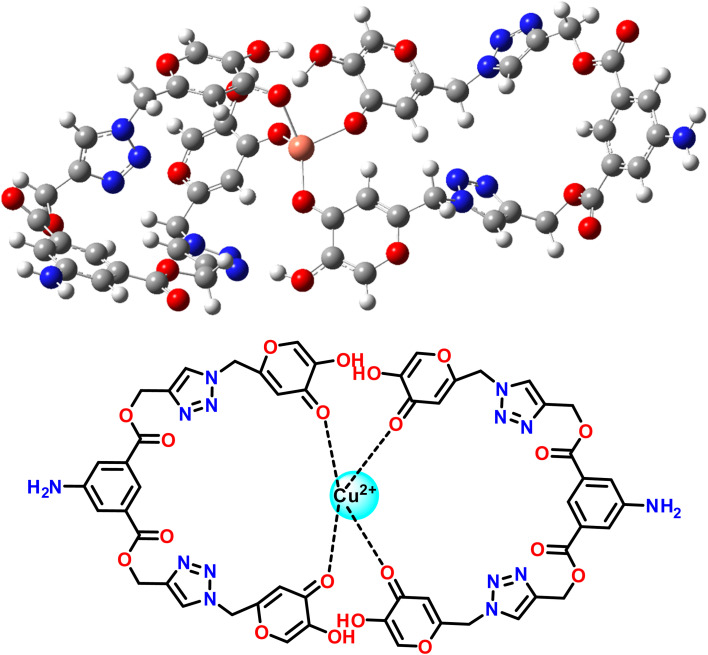
Plausible binding mode between 3 and Cu^2+^ ions.

The hard and soft acids and bases (HSAB) concept categorizes Cu^2+^ as a borderline acid.^44^ In contrast, probe 3 possessed N and O atoms with lone pairs, making them electron-rich sites capable of interacting with metal ions. Moreover, the Job plot analysis of the probe with the metal ions established a 2 : 1 L : M ratio. In light of the HSAB principles and the host–guest relationship confirmed by the Job plot analysis, a plausible binding mode is depicted in Fig. 13, illustrating the metal ion binding to the ligand through the O atoms of the probe.

## 4. Conclusion

In conclusion, we synthesized a new fluorescent chemosensor 5-aminoisophthalate and kojic acid-based 1,2,3-triazole scaffolds to recognize Cu^2+^ ions. Upon the addition of Cu^2+^ ions, an almost two-fold enhancement in the emission intensity was exhibited. From the Job's plot, the binding stoichiometries of the complex were determined to be 2 : 1 (L : M). The association constant for the formation of the Cu^2+^ complex was determined to be 4.89 × 10^3^ M^−1^. The detection limit of probe 3 was 8.82 μM, much lower than the value recommended by the EPA. The time and temperature-dependent analyses revealed that the chemosensor was stable, independent of the time and temperature upon complexation with Cu^2+^ ions. Reversibility analysis indicated that probe 3 could be used as both a reusable sensor and as a scavenger of copper ions. A comparison analysis of LODs from previously developed chemosensors for Cu^2+^ ions revealed that our probe is the most potent among them. DFT studies confirmed the complex formation mechanisms. Hirshfeld surface analysis revealed significant intermolecular interactions in compound 1. Energy framework calculations indicated favorable interactions for the title compound 1. Molecular docking was performed to investigate the interaction between the probe and selected bacterial target with a B.E. of −9.2 kcal mol^−1^. This dual-purpose investigation sheds light on the probe's ability to selectively detect Cu^2+^ ions and its potential as an antibacterial agent.

## Author contributions

Sachin Kumar: methodology, investigation, validation, writing manuscript. Bajrang Lal: methodology, investigation, validation. Gurleen Singh: validation, writing manuscript. Muskan: crystallographer. Ram Kumar Tittal: supervision, resources, writing: review and editing. Jandeep Singh: conceptualization, resources. Vikas D. Ghule: software. Renu Sharma: supervision.

## Conflicts of interest

The authors declare no conflicts of interest.

## Supplementary Material

RA-014-D4RA02372B-s001

RA-014-D4RA02372B-s002
