# Supplementary material for: 5-Aminoisophthalate-based kojic acid-appended bis-1,2,3-triazole: a fluorescent chemosensor for Cu2+ sensing and in silico study
Source: RSC Adv. 2024 Jul 3;14(29):20908–22. doi: 10.1039/d4ra02372b (PMC11220489; doi:10.1039/d4ra02372b)
Supplement: RA-014-D4RA02372B-s001 [file RA-014-D4RA02372B-s001.pdf]

Electronic Supplementary Material (ESI) for RSC Advances.
This journal is © The Royal Society of Chemistry 2024

5-Aminoisophthalate based Kojic Acid appended Bis-1,2,3-Triazole: A Fluorescent Chemosensor for Cu^{2+} Sensing & *In Silico* Study

Sachin Kumar[1,2] Ram Kumar Tittal,[1,3,*] Gurleen Singh,[4] Muskan,[5] Ghule Vikas D.,[1] Renu Sharma[2] Jandeep Singh,[4,#]

[1] Department of Chemistry, National Institute of Technology, Kurukshetra, Haryana 136119, India
[2] School of Applied Sciences, Om Sterling Global University, Hisar, Haryana 125001, India
[3] Department of Chemistry, Harcourt Butler Technical University, Kanpur-208002, U.P., India. (Present address)
[4] School of Chemical Engineering and Physical Sciences, Lovely Professional University, Phagwara, Punjab 144411, India
[5] Department of Chemistry, University of Delhi, Delhi 110007, India

*,# Corresponding authors

* rktittaliitd@nitkkr.ac.in; Ph: +91-1744-233-542; # singhjandeep@gmail.com

Table of contents:

S.No.	Contents	Page No.
1.	X-Ray Crystallography Experimental Section	S-2
2.	Table SI-1. Crystal data and structure refinement for **1**.	S-2 to S-3
3.	Figure SI-1, Unit cell showing four molecular units in the asymmetric unit of compound **1**	S-3
4.	Figure SI 2-5, Physical data (FTIR, ^{1}H-NMR, ^{13}C-NMR, HRMS of **1**)	S-4 to S-5
5.	Figure SI 6-9, Physical data (FTIR, ^{1}H-NMR, ^{13}C-NMR, HRMS of **2**)	S-6 to S-7
6.	Figure SI 10-13, Physical data (FTIR, ^{1}H-NMR, ^{13}C-NMR, HRMS of probe **3**)	S-8 to S-9
7.	Figure SI-14, B-H plot for the complexation between probe **3** and Cu^{2+} ions.	S-10
8.	Figure SI-15, Absorption spectra of **3.Cu^{2+}** complex solution showing the independence of absorption intensity of time	S-10
9.	Figure SI-16, The absorption spectra of **3.Cu^{2+}** solution, after being subjected to a temperature range of 20-50°C	S-11
10.	Figure SI-17, Change in absorption of the **3.Cu^{2+}** complex upon the addition of EDTA.	S-11
11.	Figure SI-18, Correlation plot of an emission spectrum of probe **3** with Cu^{2+} molar concentration (μM).	S-12
12.	Figure SI-19, Optimized structures of **1**, **2** and **3** with B3LYP/6-311G (d,p) level of theory via the Gaussian 09 package and their Contour plots.	S-12
13.	Table SI-2. Docking results of probe **3** docked in the active site of *E. coli* DNA gyrase topoisomerase II	S-13
14.	Table SI-3. Docking results of probe **3** docked in the active site of cytochrome P450 14α-sterol demethylase	S-13
15.	Figure SI-20. 2D and 3D interactions of probe **3** docked in the active site of cytochrome P450 14α-sterol demethylase.	S-13
16.	References	S-14

X-Ray Crystallography Experimental Section:

A needle shaped clear light colourless single crystals were carefully picked under a polarizing microscope and pasted to an excellent fine glass fiber with the help of cyanoacrylate (superglue) adhesive. The single-crystal X-ray diffraction data were collected with 'Oxford XCalibur CCD diffractometer with monochromatic Mo *Kα* radiation (λ = 0.71073 Å) at 296.15 K by using ω and ϕ *s*can. The X-ray generator was operated at 50 kV and 20 mA. The data were reduced by using APEX3, the SAINT V8.40B program and was used for diffraction profiles integration. The structure was solved and refined by the full-matrix least square method on F^2 using the SHELXL 2018/3 & SHELXT 2018/2 program respectively[1,2], present in the Olex2 package of programs (version 1.3.0)[3]. All the hydrogen positions were initially located in the different Fourier maps. For the final refinement, the hydrogen atoms were placed in geometrically ideal positions and refined in the riding mode. The final refinement comprised the atomic positions of all the atoms, isotropic thermal parameters for all the hydrogen atoms, and anisotropic thermal parameters for all the non-hydrogen atoms. Please refer to the following **Table SI-1** for a detailed explanation of the crystal structure solution and final refinements for the structures. The crystallographic data for compound **1** can be found in CCDC No: **2300460** free of charge from The Cambridge Crystallographic Data Centre (CCDC) via www.ccdc.cam.ac.uk/ data_request/cif.

Table SI-1 Crystallographic data and structure refinement for compound **1.**

Identification code	**1**
Crystal structure	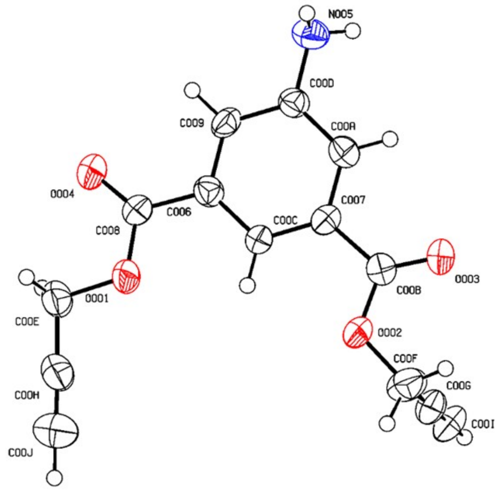
Empirical formula	$C_{13.75} H_{10.75} N O_4$
Formula weight	253.90
Temperature	216 K

Wavelength	0.71073 Å	
Crystal system	Monoclinic	
Space group	P 21/c	
Unit cell dimensions	a = 4.7711(6)	α = 90
	b = 13.430 (5)	β = 94.722(10)
	c = 19.544 (2)	γ = 90
Volume	1248.1(5) $Å^3$	
Z	4	
Density (calculated)	1.352 g/cm^3	
Absorption coefficient	0.101 mm^{-1}	
F(000)	529.0	
Index ranges	h= 5, k = 11, l = 22	
Reflections collected	0.0567(807)	
Completeness to theta = 26.37 °	99.7%	
Final R indices [I>2 sigma(I)][a,b]	R1 = 0.0567, wR2 = 0.1805	

[a] $R = \sum(\|Fo| - |Fc\|)/\sum|Fo|$; [b] $Rw = \{\sum[w(Fo^2 - Fc^2)^2]/\sum[w(Fo^2)^2]\}^{1/2}$

The X-ray single-crystal structure study revealed that compound **1** was shaped in a monoclinic crystal system. Compound **1** crystallizes in monoclinic cells with space group 'P 21/c'. The resultant structure of compound **1** contains four molecular units in the asymmetric unit and is represented in **Figure SI-1**.

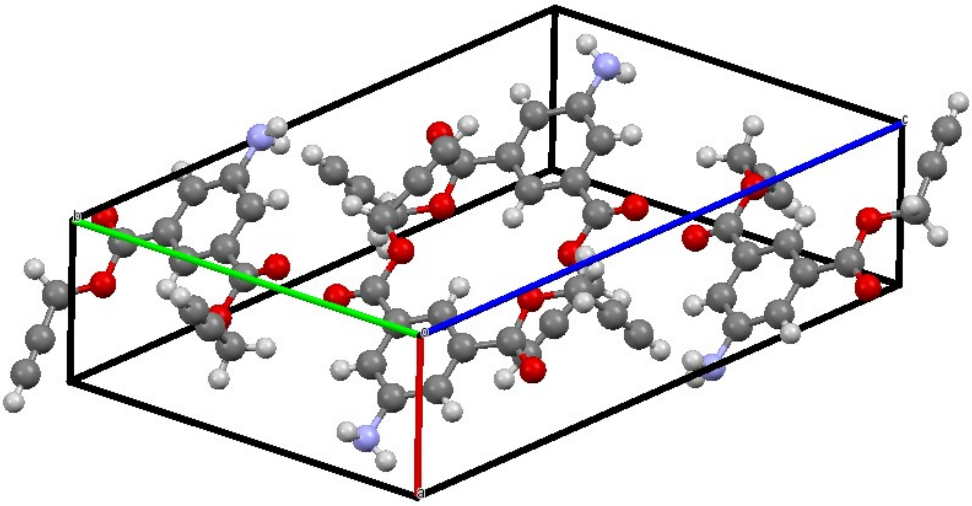

Figure SI-1. Unit cell (Z=4) showing four molecular units in the asymmetric unit of compound **1**.

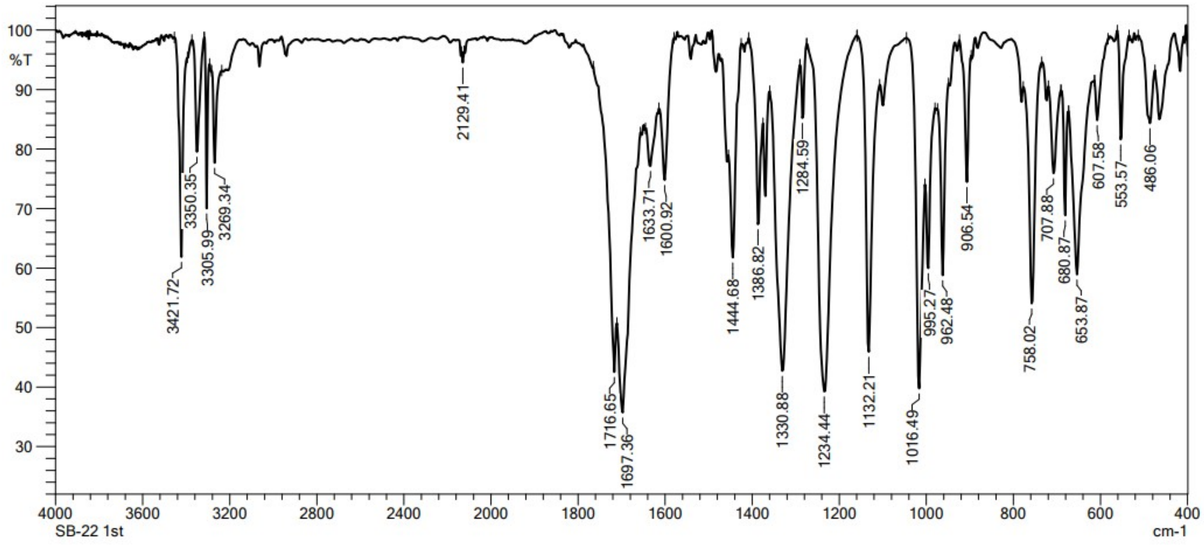

Figure SI-2. FTIR spectra of alkyne, **1**.

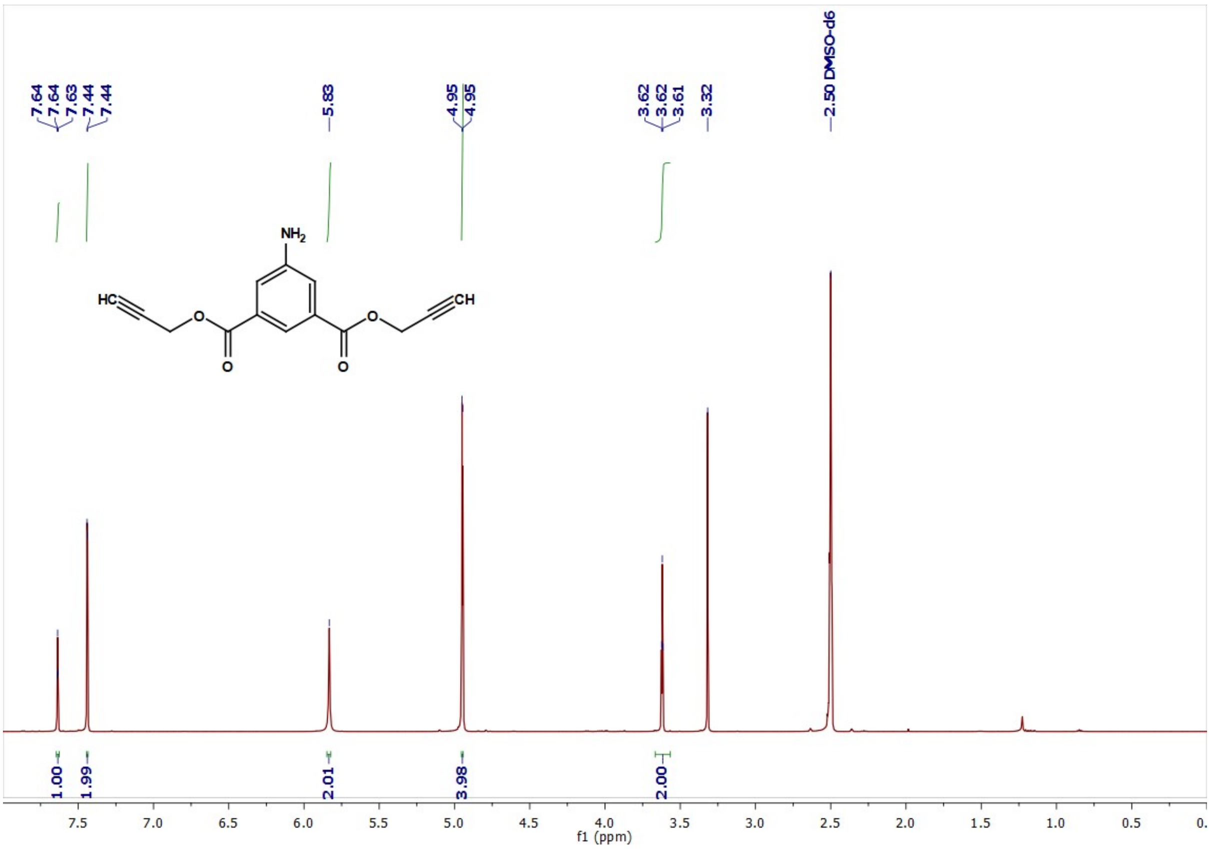

Figure SI-3. ^{1}H NMR spectra of alkyne, **1**.

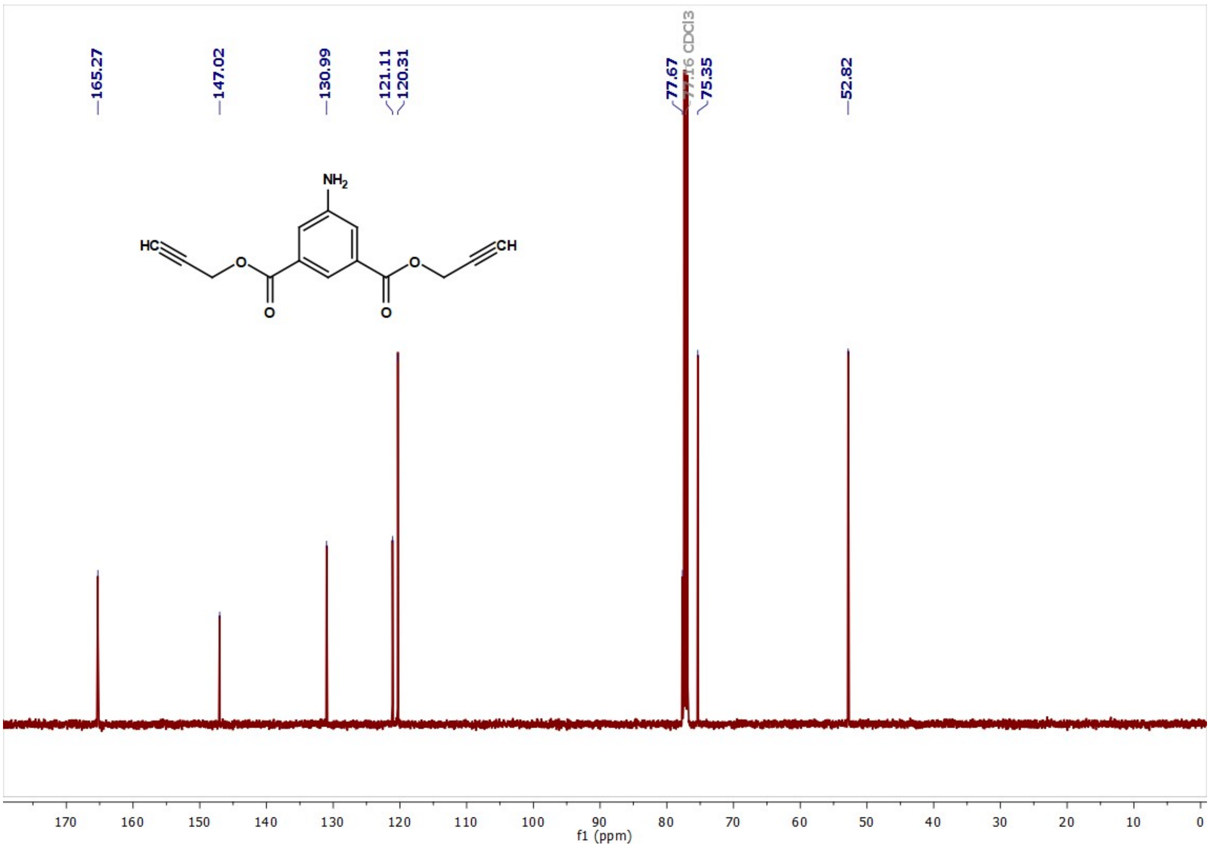

Figure SI-4. ^{13}C NMR spectra of alkyne, **1**.

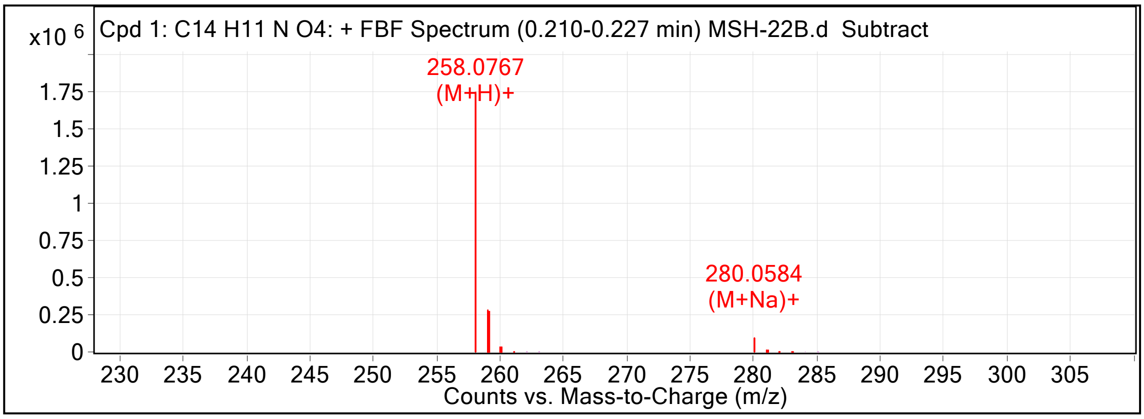

Figure SI-5. HRMS spectra of alkyne, **1**.

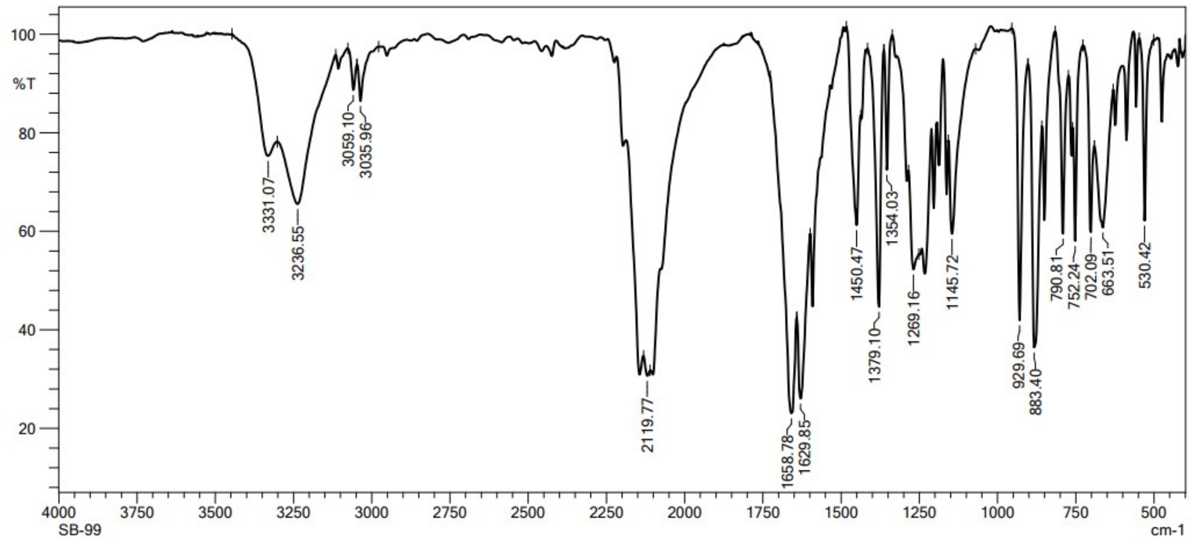

Figure SI-6. FTIR spectra of azide, **2**.

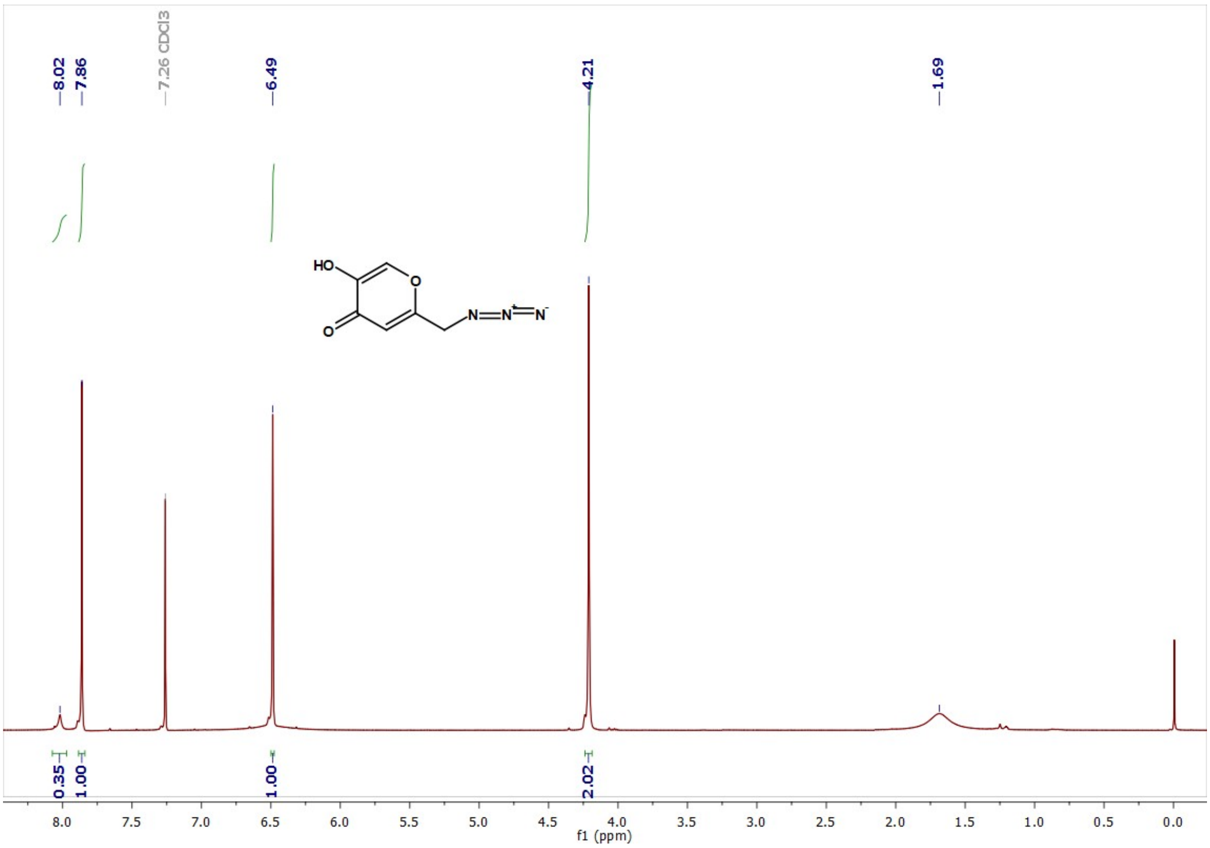

Figure SI-7. ^{1}H NMR spectra of azide, **2**.

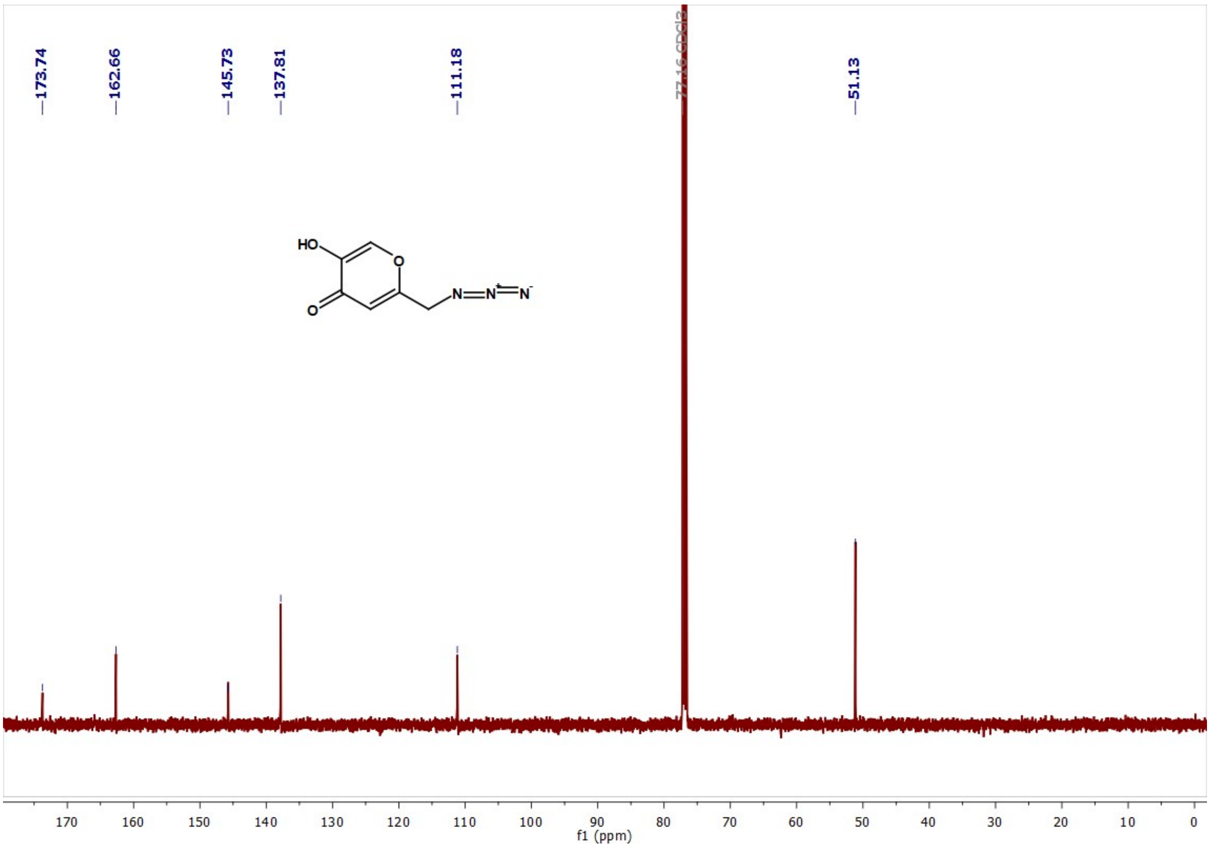

Figure SI-8. ^{13}C NMR spectra of azide, **2**.

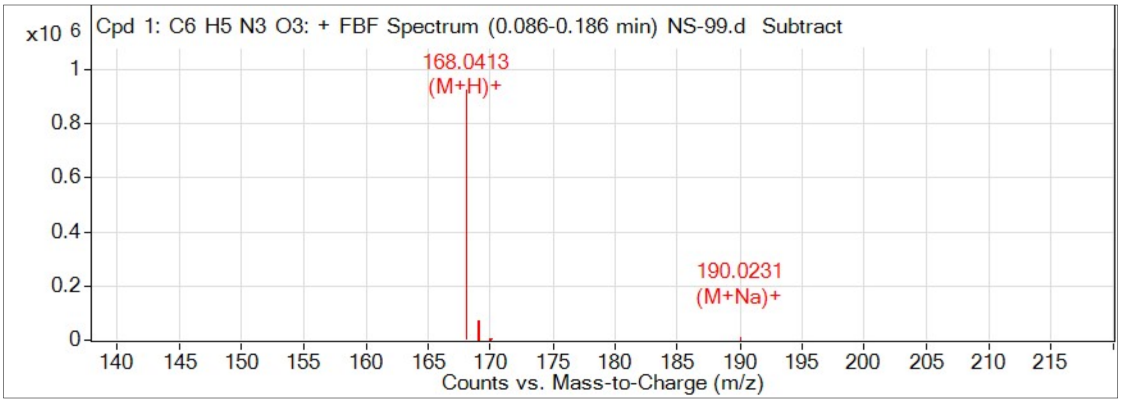

Figure SI-9. HRMS spectra of azide, **2**.

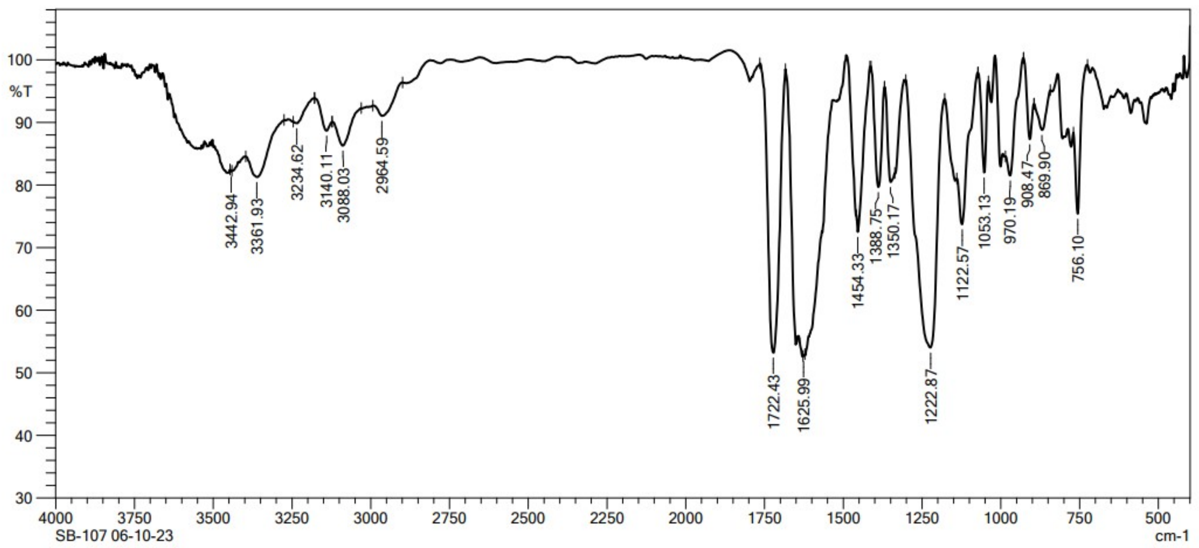

Figure SI-10. FTIR spectra of probe, **3**.

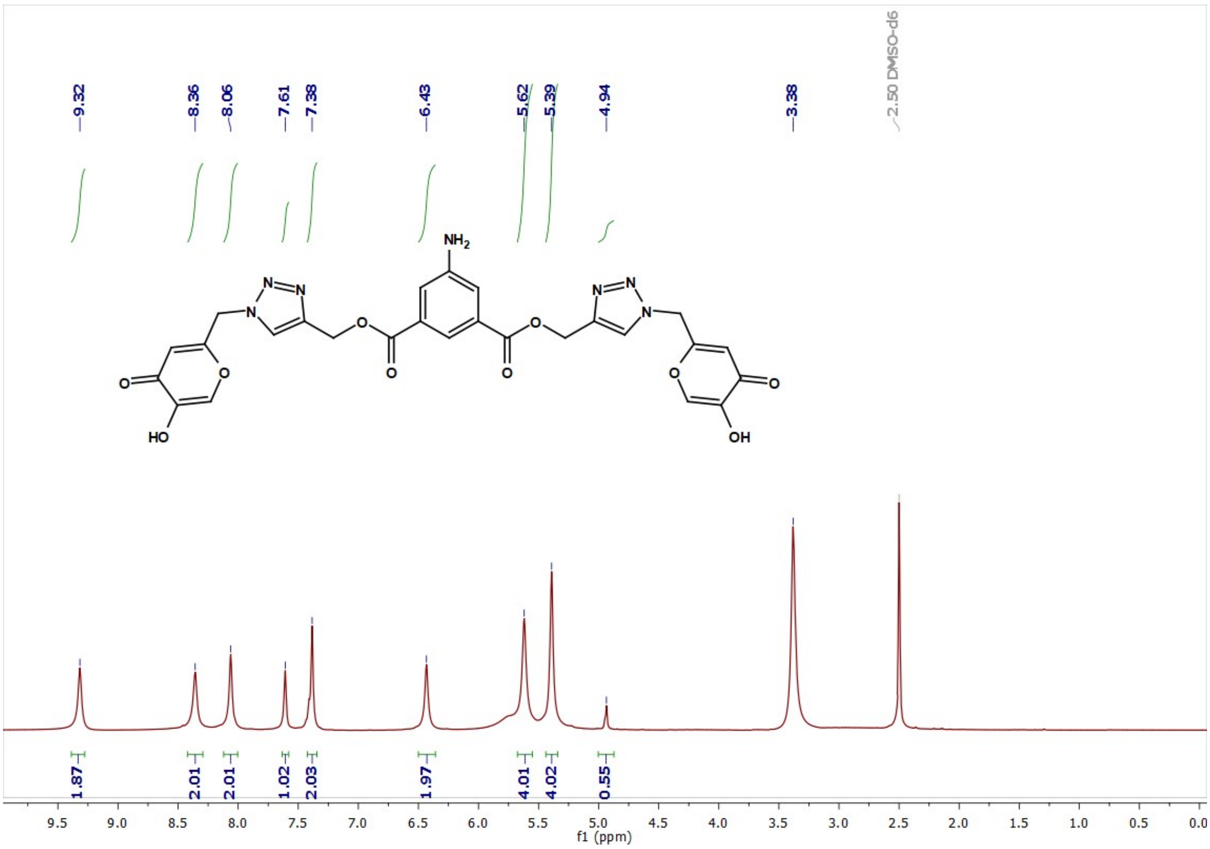

Figure SI-11. ^{1}H NMR spectra of probe, **3**.

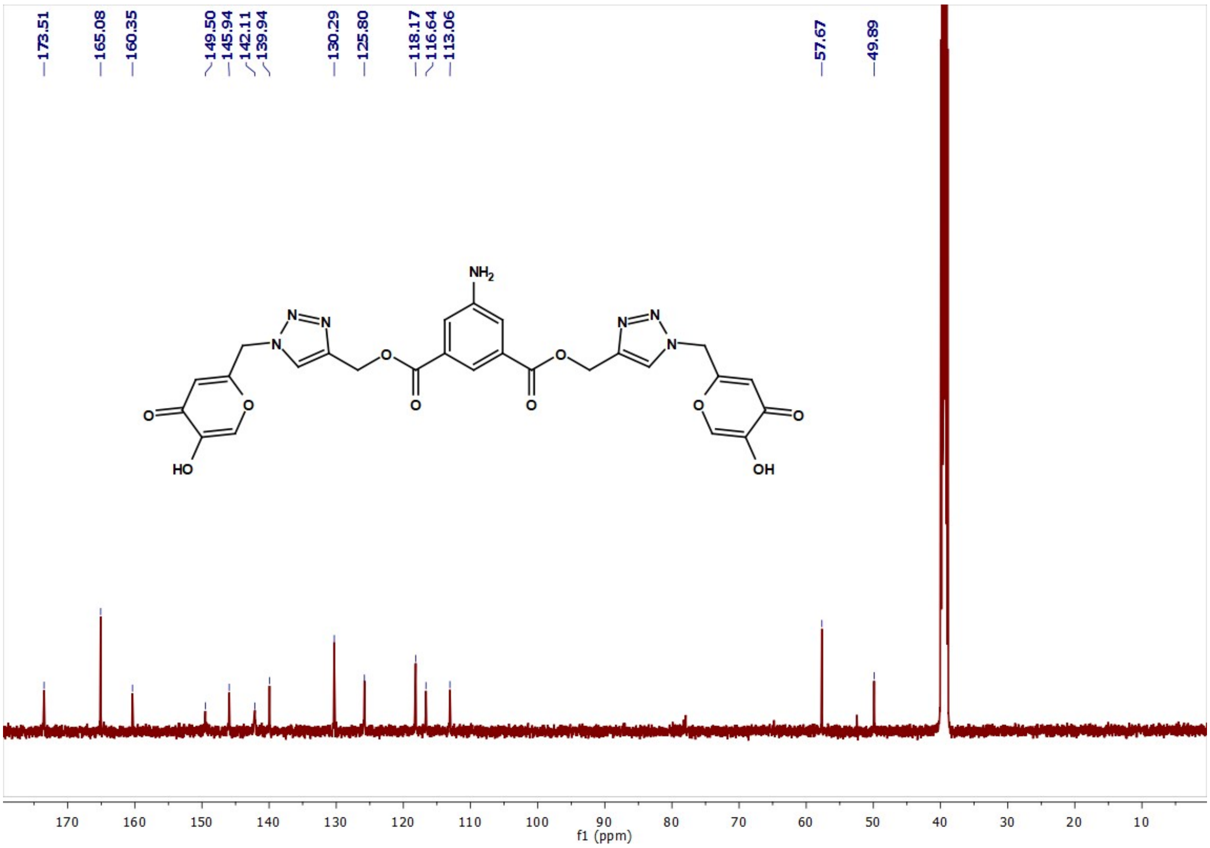

Figure SI-12. ^{13}C NMR spectra of probe, **3**.

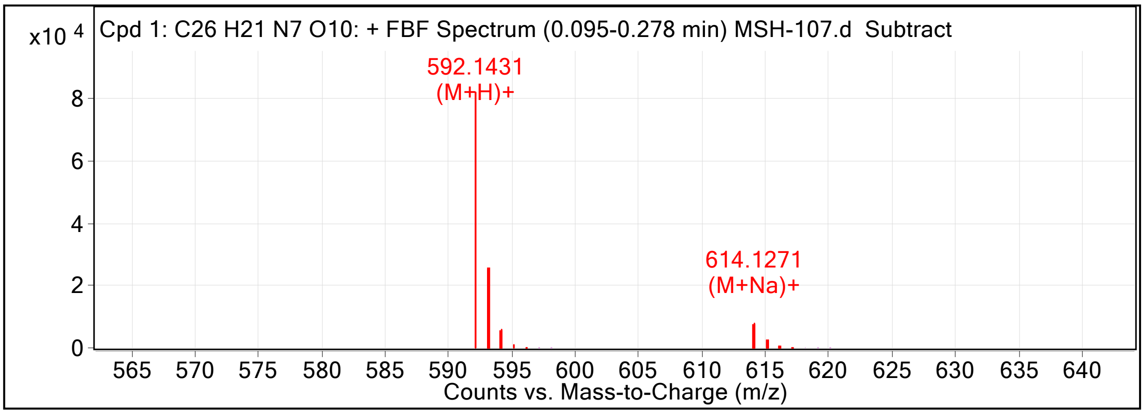

Figure SI-13. HRMS spectra of probe, **3**.

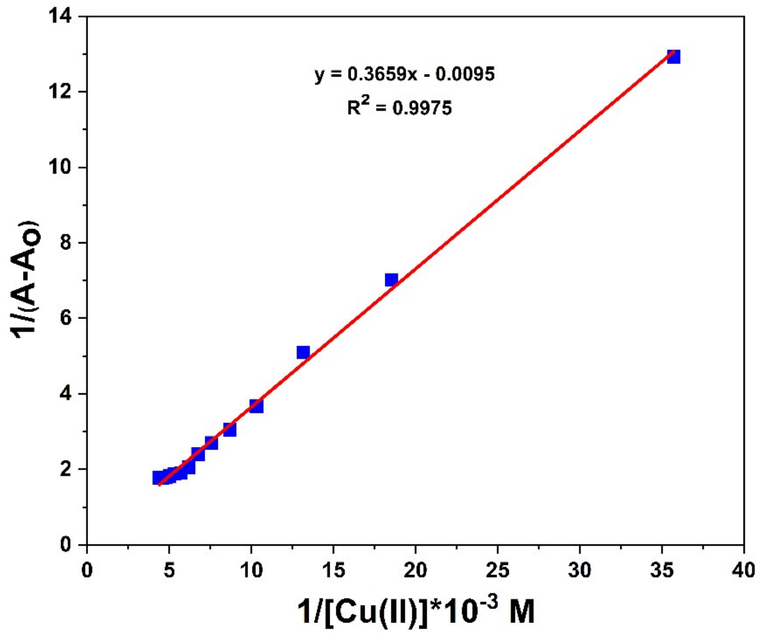

Figure SI-14. B-H plot for the complexation between probe **3** and Cu^{2+} ions.

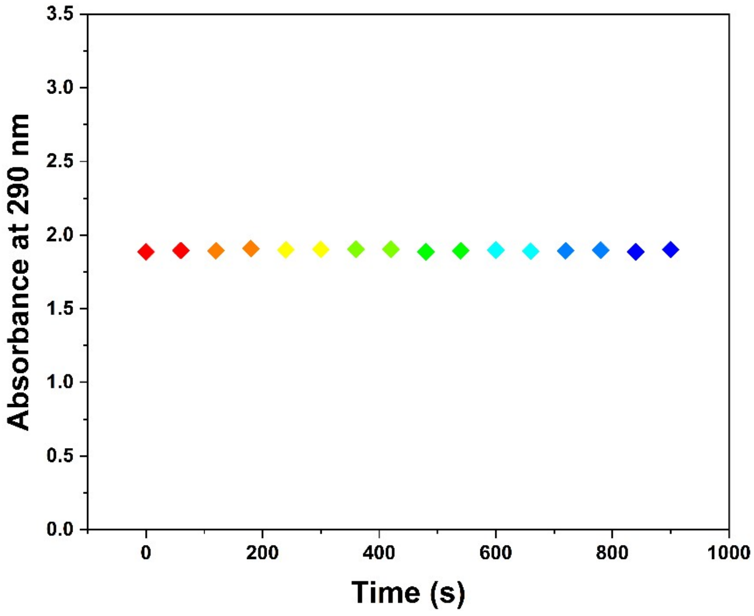

Figure SI-15. An illustration representing the absorbance over time for **3.Cu**$^{2+}$ complexation.

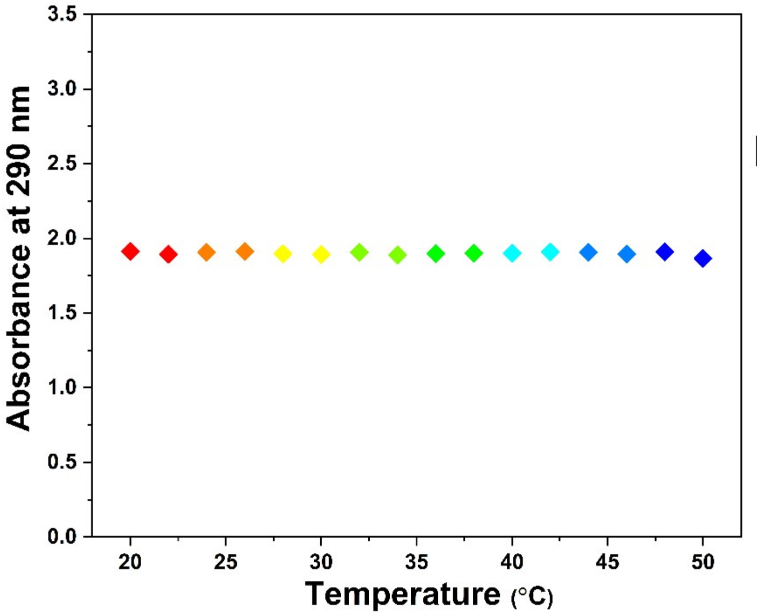

Figure SI-16. An illustration representing the absorbance over 20-50 °C temperature range for **3.Cu^{2+}** complexation.

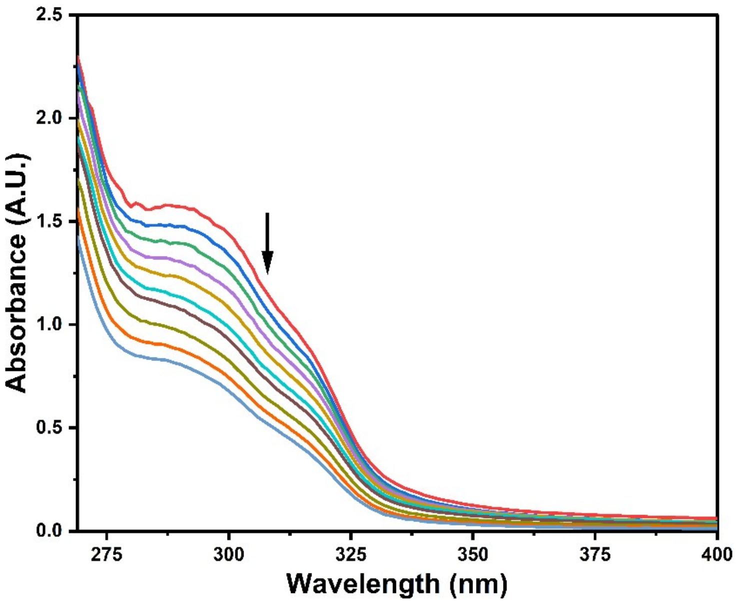

Figure SI-17. Change in absorption of the **3.Cu^{2+}** complex upon the addition of EDTA.

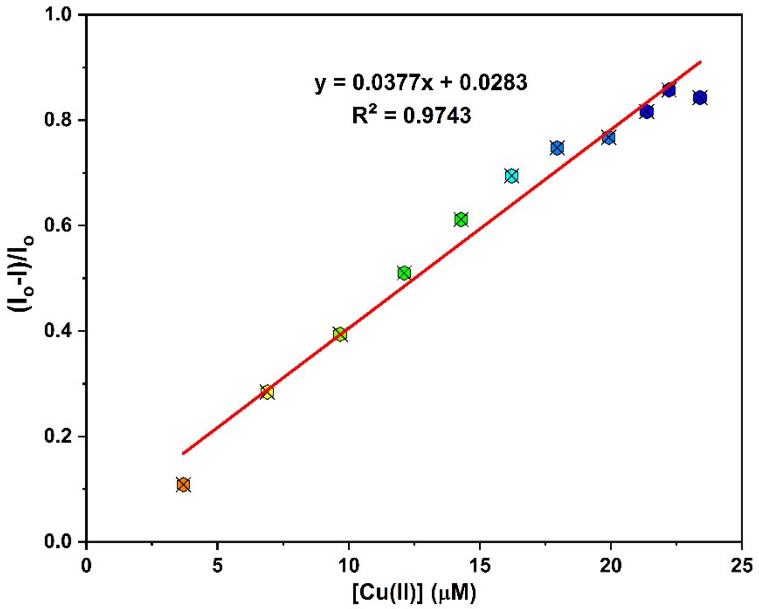

Figure SI-18. Correlation plot of an emission spectrum of probe **3** with Cu^{2+} molar concentration (μM).

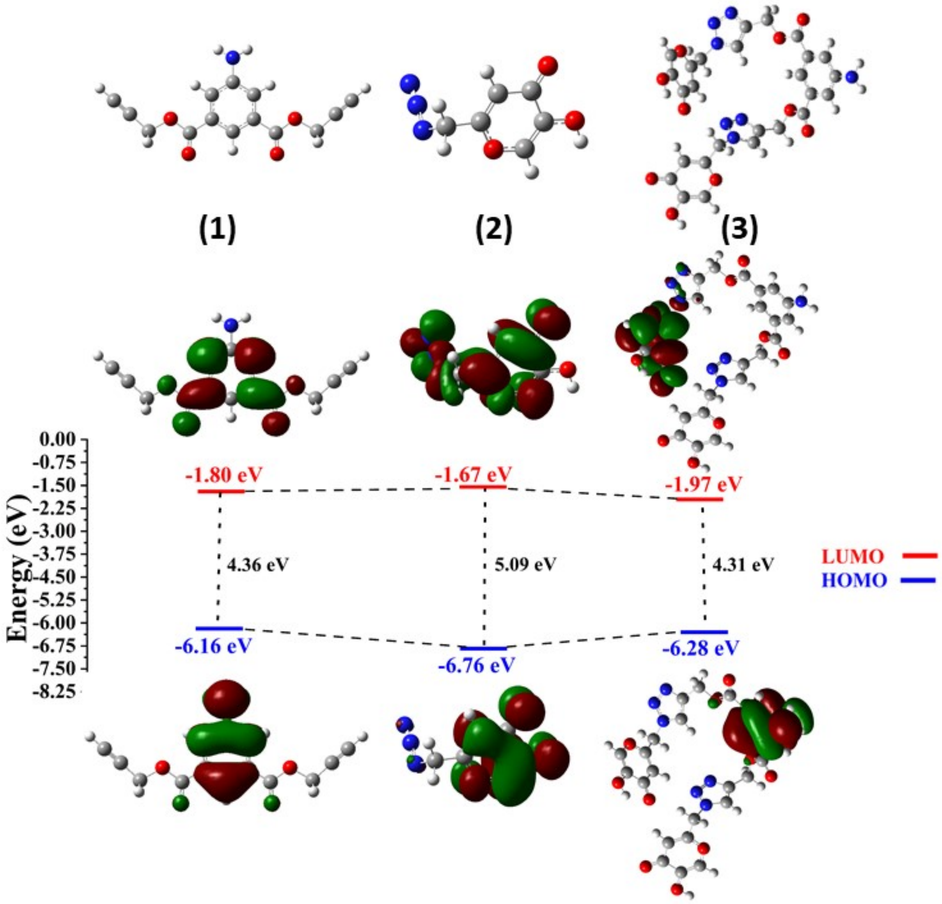

Figure SI-19. Optimized structures of **1**, **2** and **3** with B3LYP/6-311G (d,p) level of theory via the Gaussian 09 package and their Contour plots.

Table SI-2. Docking results of probe **3** docked in the active site of *E. coli* DNA gyrase topoisomerase II (PDB ID: 1KZN) by using AutoDock Vina.

Compound	H-bond interactions with bond length	Other Interactions	B.E. (kcal mol^{-1})
Probe **3**	ASN46(3.22 Å), SER121(2.80 Å), SER121(3.19 Å), VAL167(3.26 Å), VAL71(2.85 Å), ILE90(2.71 Å)	PRO79, GLU50, ASN46, VAL120, THR165, ILE78, ALA47, VAL167, ILE90	-9.2

Table SI-3. Docking results of probe **3** docked in the active site of cytochrome P450 14α-sterol demethylase (CYP51) (PDB ID: **1EA1**) by using AutoDock Vina.

Compound	H-bond interactions with bond length	Other Interactions	B.E. (kcal mol^{-1})
Probe **3**	GLN72(2.11 Å), THR260(2.51 Å), PHE255(2.90 Å), ALA256(3.30 Å), TYR76(2.90 Å), ASP67(3.19 Å)	ASP71, ALA73, PHE83, HEM460, MET79, LEU321, ALA73, ARG95	-8.8

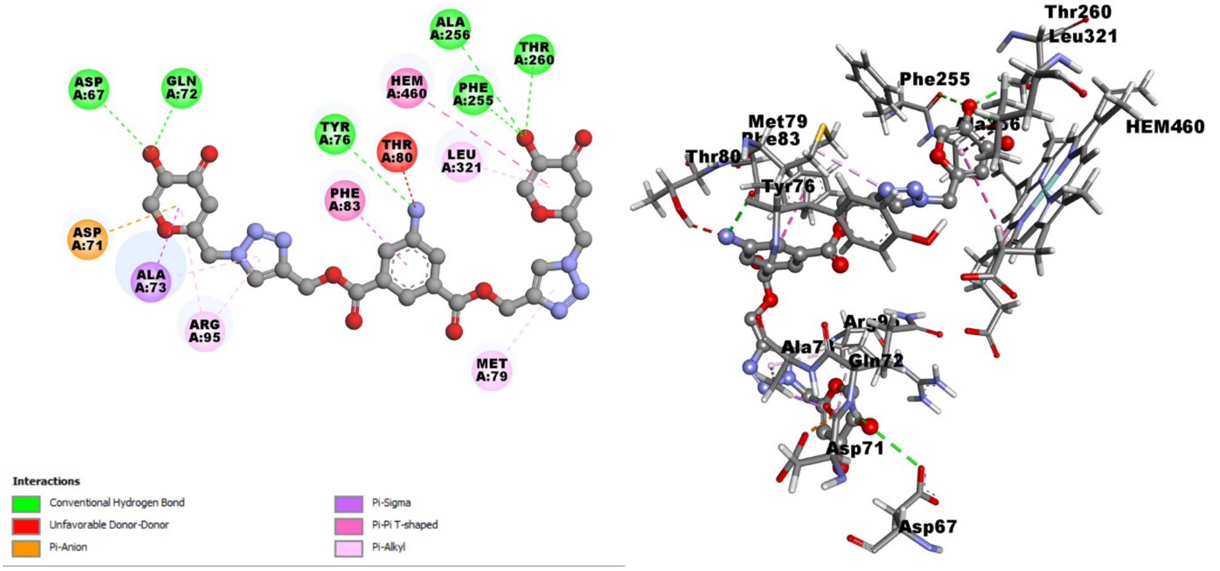

Figure SI-20. 2D and 3D interactions of probe **3** docked in the active site of cytochrome P450 14α-sterol demethylase.

References:

1 G. M. Sheldrick, *Acta Crystallogr. Sect. A*, 2015, **71**, 3–8.

2 G. M. Sheldrick, *Acta Crystallogr. Sect. C*, 2015, **71**, 3–8.

3 O. V Dolomanov, L. J. Bourhis, R. J. Gildea, J. A. K. Howard and H. Puschmann, *J. Appl. Crystallogr.*, 2009, **42**, 339–341.
